# Self-assembly and regulation of protein cages from pre-organised coiled-coil modules

**DOI:** 10.1038/s41467-021-21184-6

**Published:** 2021-02-11

**Authors:** Fabio Lapenta, Jana Aupič, Marco Vezzoli, Žiga Strmšek, Stefano Da Vela, Dmitri I. Svergun, José María Carazo, Roberto Melero, Roman Jerala

**Affiliations:** 1 Department of Synthetic Biology and Immunology, National Institute of Chemistry, Ljubljana, Slovenia; 2 EN-FIST Centre of Excellence, Ljubljana, Slovenia; 3 Department of Chemistry, Life Sciences and Environmental Sustainability, University of Parma, Parma, Italy; 4 EMBL c/o DESY, 2607 Hamburg, Germany; 5 Centro Nacional de Biotecnología (CNB-CSIC), Madrid, Spain

**Keywords:** Synthetic biology, Nanostructures, Protein design, SAXS

## Abstract

Coiled-coil protein origami (CCPO) is a modular strategy for the de novo design of polypeptide nanostructures. CCPO folds are defined by the sequential order of concatenated orthogonal coiled-coil (CC) dimer-forming peptides, where a single-chain protein is programmed to fold into a polyhedral cage. Self-assembly of CC-based nanostructures from several chains, similarly as in DNA nanotechnology, could facilitate the design of more complex assemblies and the introduction of functionalities. Here, we show the design of a de novo triangular bipyramid fold comprising 18 CC-forming segments and define the strategy for the two-chain self-assembly of the bipyramidal cage from asymmetric and pseudo-symmetric pre-organised structural modules. In addition, by introducing a protease cleavage site and masking the interfacial CC-forming segments in the two-chain bipyramidal cage, we devise a proteolysis-mediated conformational switch. This strategy could be extended to other modular protein folds, facilitating the construction of dynamic multi-chain CC-based complexes.

## Abstract (web summary)

Coiled-coil protein origami is a strategy for the de novo design of polypeptide nanostructures based on coiled-coil dimer forming peptides, where a single chain protein folds into a polyhedral cage. Here, the authors design a single-chain triangular bipyramid and also demonstrate that the bipyramid can be self-assembled as a heterodimeric complex, comprising pre-defined subunits.

## Introduction

Tailor-made nanostructures enable precise control over three-dimensional spatial arrangements and biochemical processes at the molecular level. Biological macromolecules, such as DNA and polypeptides, represent versatile, programmable biomaterials suitable for this purpose. Both DNA nanotechnology and de novo protein design are currently experiencing an extraordinary expansion in terms of diversity and complexity of designable nano- and microscale architectures^1,2^. In designing nanostructures, modularity is a commonly employed concept since it greatly simplifies the design process. Based on the structure and interaction patterns of modular building units, it is possible to design either large single-chain or multimeric protein complexes^3–6^. Novel protein complexes have been obtained via the fusion of either naturally occurring^7,8^ or de novo designed^9–11^ oligomerising domains, by the de novo design of protein–protein interfaces^12–14^ or by designing metal-mediated interactions^15–17^. In contrast, DNA nanotechnology relies primarily on the application of the modular and discrete base-pairing^18^ and base-stacking^19,20^ rules offered by the DNA double helix, enabling the design and construction of high-order structures, switches and dynamic assemblies^21–23^.

Translating the modular paradigm of DNA nanotechnology to the protein realm is achievable by employing α-helical elements as building modules. Their specificity of the interaction, small size and the discrete rules governing their oligomerisation properties make α-helical elements highly versatile building blocks for protein design^24–31^. This is particularly true in the case of coiled-coil (CC) units. CCs are super-secondary structural elements ubiquitous in every domain of life^32,33^ and have been widely used as protein recruitment domains both in vitro^34–40^ and in vivo^41–44^. Geometric protein assemblies and polyhedral protein cages have been built using orthogonal interacting CC units^36,37,45,46^. Coiled-coil protein origami (CCPO) represents a type of modular design based on pairwise-interacting CC units. This strategy guides polypeptide chains to fold into polyhedral cages with internal cavities^45,46^. In our previous work, we showed that single-chain CCPO polyhedral cages, such as tetrahedra, square pyramids and trigonal prism cages, can self-assemble during translation^46^. However, larger, dynamic, altogether more versatile CC-based nanostructures might be obtained more easily by the bottom-up self-assembly of multiple pre-organised subunits, enabling, for instance, the use of the same building modules in each subunit. Similarly, DNA nanostructures assembled bottom-up from multiple complementary chains allowed the design of high-order supramolecular complexes^20,47^; however, the assembly of modular structures based on polypeptide chains is generally more demanding than using nucleic acids. If successfully applied to CCPO assemblies, bottom-up oligomeric self-assembly could allow designing larger cages based on a given orthogonal set, since orthogonality requirements would need to be satisfied only within each independent subunit. Furthermore, elucidating the rules governing the oligomeric self-assembly of CCPO cages could facilitate the design of nanostructures with more complex topologies and introduce functionalities such as conformational regulation and responsiveness to external cues, such as proteolytic activity.

Here, we investigated whether CCPO cages could be generated as oligomeric assemblies. First, a single-chain triangular CCPO bipyramid, representing a de novo designed polyhedral protein fold comprising 18 CC-forming segments, was designed and characterised. Next, the cage was re-designed as a heterodimeric complex consisting of a larger pre-organised subunit and a short unstructured peptide or from two pre-organised tetrahedral subunits, showcasing the implementation of a bottom-up self-assembly strategy in a de novo designed CCPO cage. Furthermore, by incorporating a protease cleavage site in the heterodimeric CCPO bipyramid, we obtained a conformational switch controlled by proteolysis, demonstrating that polyhedral protein cages can be designed to transition between two structural states in response to external cues.

## Results

### Construction of a single-chain trigonal bipyramid CCPO cage

**Fig. 1:**
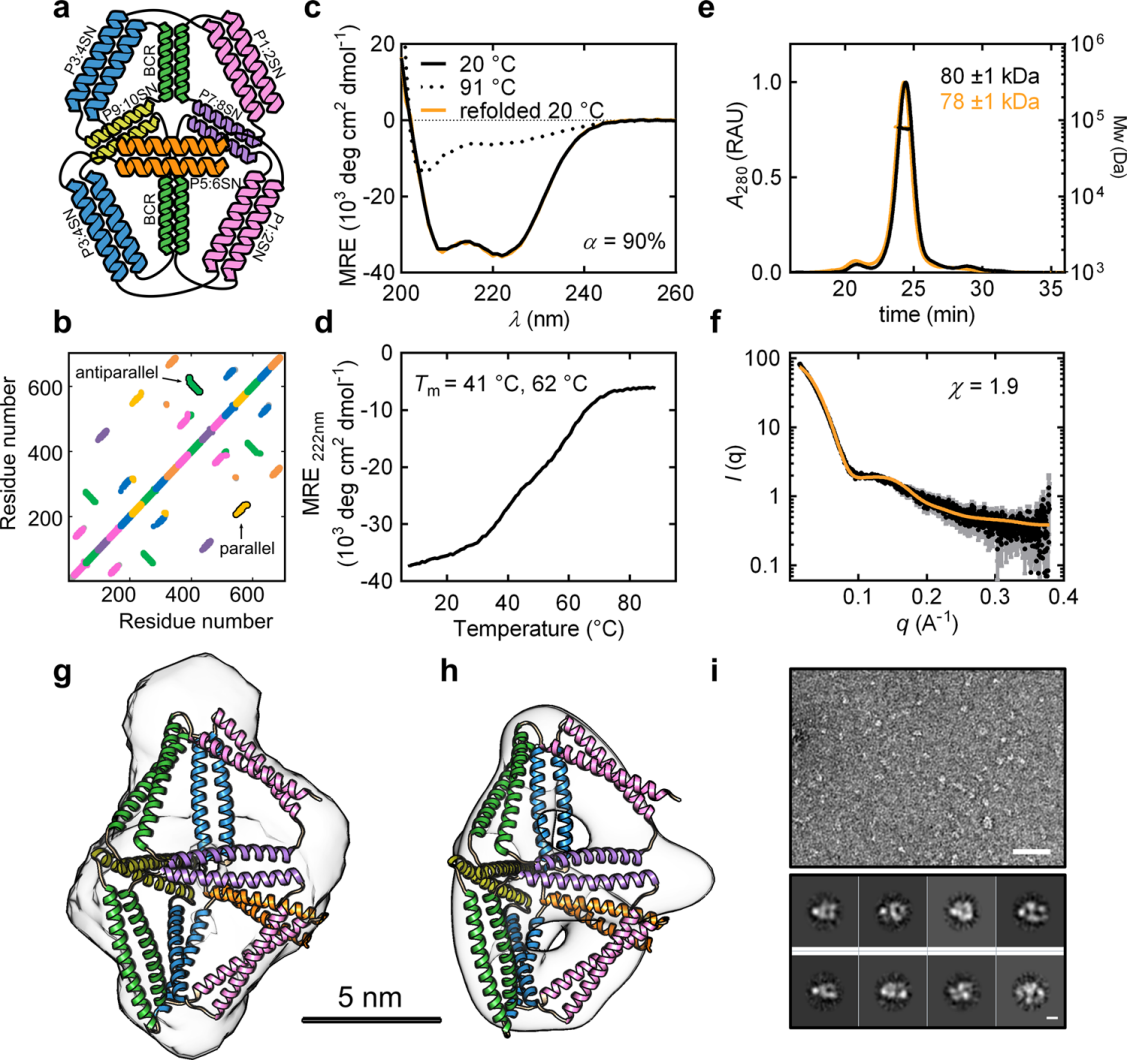
Design and characterisation of the single-chain CCPO trigonal bipyramid cage BIP18SN. **a** Topological scheme of BIP18SN; CC pairs are represented as coloured helices. **b** Contact map of amino acids (8 Å distance cut-off) in the model of BIP18SN shown in **g** and **h**. Representative parallel and antiparallel CC dimers are indicated. **c** Circular dichroism (CD) spectra of the protein BIP18SN at 20 °C, 91 °C and 20 °C after refolding. **d** CD signal at 222 nm expressed in mean residue ellipticity (MRE) of the protein BIP18SN during thermal denaturation, the melting temperatures (*T*_m_) are indicated in the panel. **e** SEC-MALS chromatogram of BIP18SN before and after refolding (black and orange traces, respectively). UV signal is reported in relative absorbance units (RAU). The molecular weight of the main peak calculated from light scattering is indicated in the figure and corresponds to the theoretical mass calculated from the amino acid sequence (theoretical Mw of BIP18SN = 80.0 kDa). The data are representative of three independent repetitions of the experiment (*n* = 3). **f** Experimental SAXS profile of BIP18SN (black trace) and theoretical scattering calculated for the model structure shown in panel **f** (orange trace). Error bars in grey represent the standard deviation for each data point in black (mean). **g** SAXS ab initio reconstruction superimposed on the model exhibiting the best fit to the experimental SAXS data (*χ* = 1.9). The bar indicates a distance of 5 nm. **h** Electron density calculated from the single-particle reconstruction of negative-stain TEM images overlaid with the model exhibiting the best fit to the experimental SAXS data. **i** Above, representative section of 150 negative-stain TEM micrographs of BIP18SN (scale bar = 50 nm). Below, reference-free two-dimensional (2D) class averages from negative-stain TEM micrographs of BIP18SN (scale bar = 5 nm). Source data are provided as a Source Data file.

The largest designed CCPO cage previously reported was a triangular prism composed of 18 CC-forming segments, comprising ~700 residues. While small-angle X-ray scattering (SAXS) confirmed that the cage folded into the desired shape, it also indicated the coexistence of at least two conformations in solution. This was ascribed to the structural flexibility created by the four-edged faces of the polyhedron, which can adopt a rectangular or oblique conformation^46^. We expected this heterogeneity could be avoided by the design of a polyhedron composed of exclusively trigonal faces whose internal angles were fixed by the length of the edges. Using the CoCoPOD modelling platform^46^, a trigonal bipyramid CCPO cage composed of 18 CC-forming segments was designed based on previously defined principles^46^. Briefly, a selected polyhedral shape was traced as a double Eulerian trail, and different topologies and circular permutations were scored according to their topological contact order (TCO). The permutations with lower TCO represent polypeptide chains with a shorter average distance between edge-forming modules in the primary structure and were expected to fold more efficiently^46^. In case of the single-chain bipyramid, we limited the selection to chain topologies leading to a protein fold composed of two tetrahedral halves with a pseudo-mirror symmetry, each composed of nine CC-forming segments. Next, before the construction of molecular models, orthogonal CC units^48^ were assigned to each edge of the cage using the same building modules in the two halves as they were expected to fold independently (Supplementary Fig. 1a). Three orthogonal parallel heterodimeric CC pairs were positioned at the interface of the two halves, while the remaining edges were occupied by different building blocks mirrored in the two tetrahedral halves (Fig. 1a), altogether using seven parallel and two antiparallel CC dimers (Fig. 1b and Supplementary Table 1). We estimated the probability of the polypeptide chain folding correctly using a deterministic folding model^49^. Based on the model, the selected amino acid sequence had a high probability of folding correctly regardless of the repetition of the same three CC pairs in each tetrahedral subunit of the bipyramid. The design was named BIP18SN according to a nomenclature that includes the initials of the polyhedron and the number and type of CC segments used in the design. After amino acids sequence design, bipyramidal cage models were built using rigid-body molecular dynamics and refined by homology modelling (see “Methods”).

The single-chain bipyramidal protein was expressed in *E. coli* and purified from the soluble fraction. After purification (SDS-PAGE in Supplementary Fig. 2a), the protein was characterised with circular dichroism (CD), which confirmed that the polypeptide adopted a highly helical secondary structure in solution, with a calculated helical content (α) of 90% (Fig. 1c). The loss of the helical structure during thermal denaturation experiments was monitored by measuring the ellipticity signal at 222 nm. The recorded denaturation profile was characterised by two main transitions: at 41 °C and 62 °C (Fig. 1d). Analogously to the previously described CCPO cages^46^ and other highly charged proteins^50^, BIP18SN exhibited resilience to thermal unfolding, efficiently refolding after temperature quenching (Fig. 1c). This is consistent with coiled-coils’ characteristic of reversible refolding upon mechanical^51,52^ and chemical denaturation^53^. In the context of CCPO folds, previous work showed the integrity of the N-terminal capping sequence in CC being crucial for the efficient refolding after thermal denaturation^46^. This property allowed the implementation of a purification procedure (used for all the designed proteins described in this research) involving the thermal lysis of bacteria (see “Methods”). SEC-MALS analysis confirmed the monomeric state of the protein both before and after thermal denaturation (Fig. 1e). The conformation adopted by the protein cage in solution was examined with SAXS and electron microscopy (EM). The experimental SAXS curve matched the theoretical scattering calculated from a CCPO bipyramid molecular model (*χ* = 1.9), with a maximum diameter (*D*_max_) of 12.4 ± 1.0 nm and a radius of gyration (*R*_g_) of 4.6 ± 0.2 nm (Fig. 1f, g, Supplementary Fig. 3a and Supplementary Table 2). Moreover, the ab initio SAXS reconstruction based on the pair distance distribution function confirmed these results and featured an internal cavity, which is characteristic of this type of de novo protein cage design (Fig. 1g and Supplementary Fig. 4a). To further investigate the conformation assumed by the protein cage, BIP18SN was imaged by negative-stain transmission electron microscopy (TEM). The single-particle reconstruction of the electron density map confirmed the shape of the protein and the presence of an internal cavity (Fig. 1h, i).

### Construction of the CCPO bipyramid from two chains

We turned to the design of a heterodimeric version of the 18-segment bipyramid to investigate how this fold could be reconstructed from multiple polypeptide chains as a case study for hierarchically assembled CCPO cages. Different strategies for decomposing a CCPO topology into two chains offer distinct advantages that may not be equally effective. On one hand, combining two subunits with a substantial size difference—a small peptide interacting with a larger structured scaffold—offers a platform for introducing chemically synthesised peptides and additional functional components into the folded protein cage. On the other hand, assembling two equally sized, pre-organised protein subunits into a large protein architecture could enable the introduction of features, such as dynamic conformational change, and facilitate regulation of the cage’s shape and internal cavity.

**Fig. 2:**
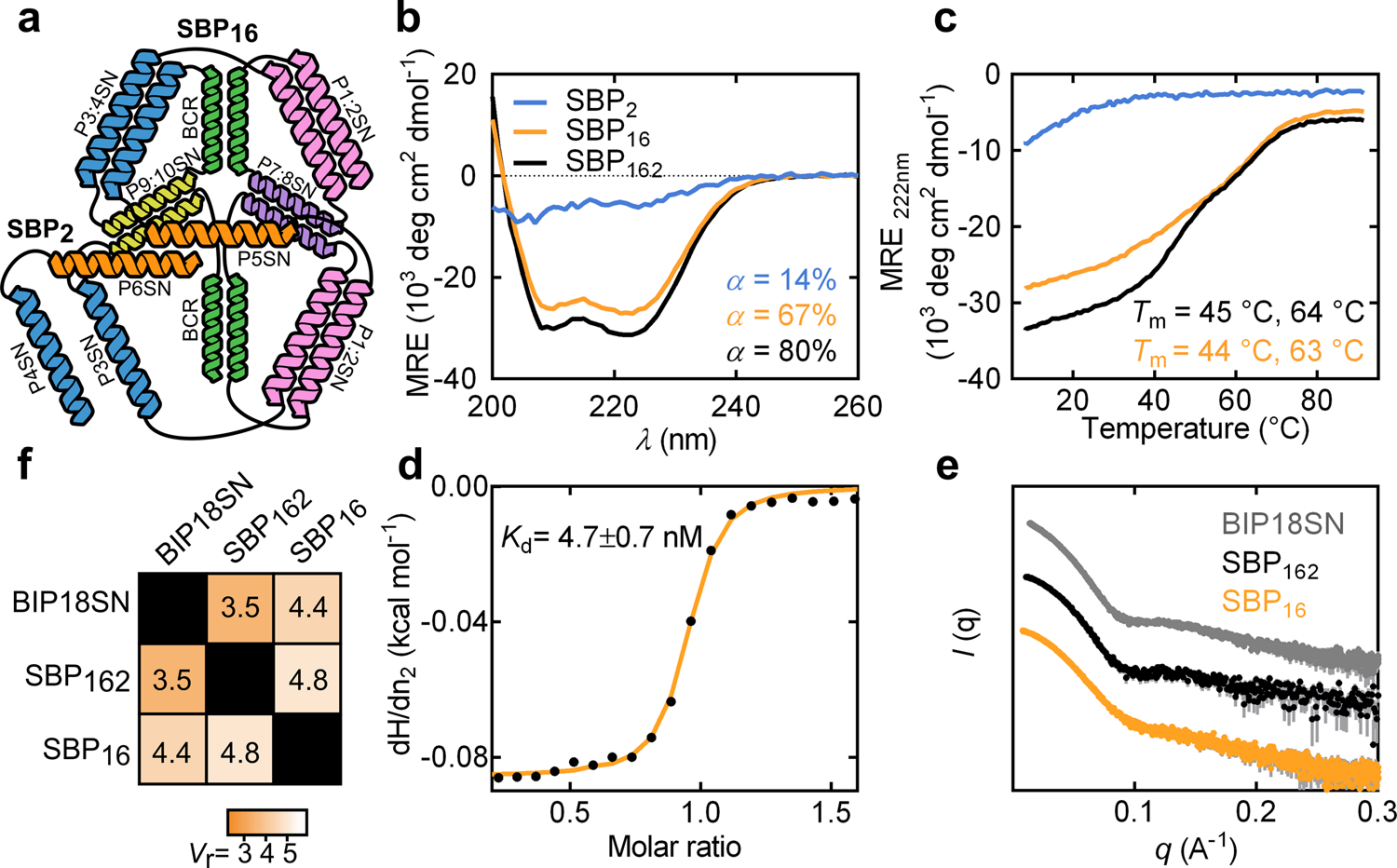
Design and characterisation of the asymmetric heterodimeric CCPO bipyramidal cage complex. **a** Topological schemes of SBP_2_ and SBP_16_; CC pairs are represented as coloured helices. **b** CD spectra of the proteins SBP_2_, SBP_16_ and the complex SBP_162_ resulting from their interaction (cyan, orange and black, respectively) at 20 °C. **c** CD signal at 222 nm of the proteins SBP_2_, SBP_16_ and the complex SBP_162_ (cyan, orange and black, respectively) during thermal denaturation, the melting temperatures (*T*_m_) are indicated in the panel. **d** ITC trace obtained by titrating SBP_16_ with SBP_2_ fitted to a 1:1 binding model (*K*_d_ = 4.7 ± 0.7 nM). **e** SAXS experimental profile of the single-chain BIP18SN protein, the complex SBP_162_ and the subunit SBP_16_ (grey, black and orange traces, respectively). Error bars in grey represent the standard deviation for each data point (mean). **f** *V*_r_ matrix comparing SAXS profiles obtained for the single-chain BIP18SN protein, the complex SBP_162_ and the subunit SBP_16_. Source data are provided as a Source Data file.

First, we tested an asymmetric deconstruction of the bipyramid into two chains of different length by trimming the two C-terminal CC-forming segments (P4SN-P6SN). The two resulting protein subunits, composed of 2 and 16 CC-forming segments, were named SBP_2_ and SBP_16_, respectively (Fig. 2a). The two proteins were separately produced in *E. coli*, purified (SDS-PAGE in Supplementary Fig. 2a) and then characterised both separately and in combination. CD analysis revealed that the larger subunit assumed a pronounced helical conformation in solution, whereas the shorter subunit showed a lack of secondary structure (Fig. 2b). The negative mean residue ellipticity (MRE) measured at 222 nm increased upon mixing the two subunits at equimolar ratio (*α* = 14 and 67% for monomeric subunits to *α* = 80% for the complex), indicating a gain in secondary structure upon binding (Fig. 2b). Analogously, thermal denaturation experiments showed the thermal stability profile of the SBP_162_ complex to be comparable to the profile observed for the single-chain BIP18SN protein (Fig. 2c). Isothermal titration calorimetry (ITC) experiments revealed a strong affinity between the two subunits (*K*_d_ = 4.7 ± 0.7 nM) and a 1:1 stoichiometry of binding (Fig. 2d and Supplementary Fig. 5a). The complex resulting from the interaction of SBP_2_ and SBP_16_, named SBP_162_, was characterised by SAXS, which confirmed that the heterodimer assumed the intended bipyramidal conformation in solution, like the single-chain bipyramid variant, with a *D*_max_ of 13.5 ± 1.0 nm and a *R*_g_ of 4.1 ± 0.1 nm (Fig. 2e, Supplementary Fig. 3a–c and Supplementary Table 2). The similarity of experimental SAXS profiles was quantitatively assessed using the volatility ratio (*V*_r_)^54^. This metric is obtained by taking the ratio of two SAXS profiles and calculating its deviation from a constant value (see “Methods”), with lower values indicating better agreement. *V*_r_ has been demonstrated to be a suitable metric for tracking conformational differences and sensitive to differences at both high and low *q*-values^55^. The *V*_r_ calculated from SAXS profiles for BIP18SN and the complex SBP_162_ of 3.5 indicated high structural similarity between the two proteins (Fig. 2e, f and Supplementary Fig. 3b, c). However, the SAXS scattering profile of the 16 CC-forming segments subunit alone showed that SBP_16_ had already adopted a conformation in close similarity to the one observed for the SBP_162_ complex (Supplementary Fig. 3a–d and Supplementary Table 2). Specifically, the relatively low *V*_r_ values between SBP_16_ and the complex SBP_162_ (*V*_r_ = 4.8) indicated the absence of a significant conformational rearrangement associated with the binding event (Fig. 2e, f and Supplementary Fig. 3b–d).

Next, aiming to extend this principle of two-chain assembly, we designed a bipyramidal CCPO complex composed of two subunits consisting of 3 and 15 CC-forming peptides (Supplementary Fig. 6). The latter, however, could not be properly characterised due to low solubility, which suggested that non-paired CC segments in a large CC-based protein might be prone to aggregation.

**Fig. 3:**
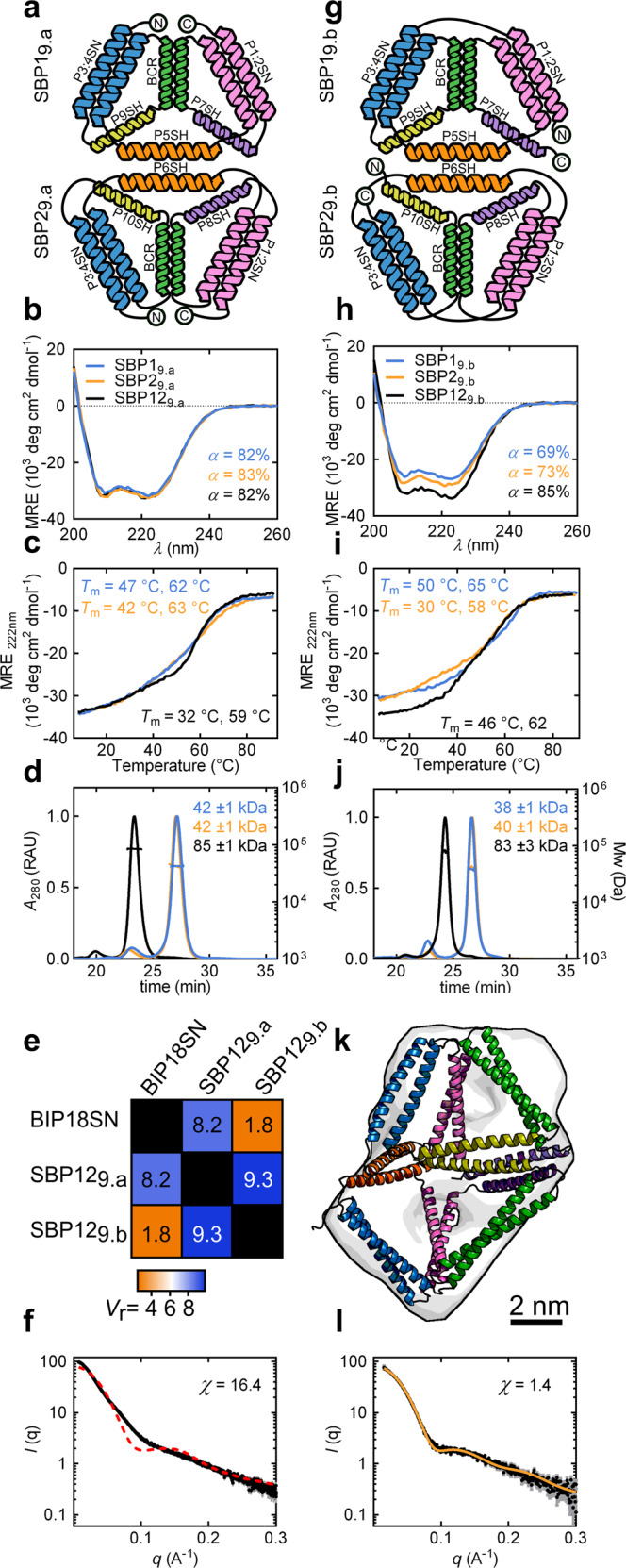
Design and characterisation of the CCPO trigonal bipyramid cage from pseudo-symmetric pre-organised subunits. **a** Topological schemes of SBP1_9.a_ and SBP2_9.a_. Coiled-coil pairs are represented as coloured helices, N- and C-termini are indicated with circled letters. **b** CD spectra of the proteins SBP1_9.a_ and SBP2_9.a_ and the complex SBP12_9.a_ resulting from their interaction (cyan, orange and black, respectively) at 20 °C. **c** CD signal at 222 nm of the proteins SBP1_9.a_ and SBP2_9.a_ and the complex SBP12_9.a_ (cyan, orange and black, respectively) during thermal denaturation, the melting temperatures (*T*_m_) are indicated in the panel. **d** SEC-MALS chromatograms and molecular masses for the proteins SBP1_9.a_ and SBP2_9.a_ and the complex SBP12_9.a_. Theoretical Mw(SBP1_9.a_) = 41.8 kDa and Mw(SBP2_9.a_) = 41.7 kDa. UV signal is reported in relative absorbance units (RAU). **e** SAXS similarity matrix for BIP18SN, the complex SBP12_9.a_ and the complex SBP12_9.b_. The similarity of conformations based on SASX results evaluated using the volatility ratio (*V*_r_) metric. **f** Comparison of the experimental SAXS profile of the complex SBP12_9.a_ (black trace) with the theoretical scattering profile calculated for the BIP18SN model structure (dotted red trace) showing the difference from the single-chain protein BIP18SN. Error bars in grey represent the standard deviation for each data point in black (mean). **g** Topological schemes of SBP1_9.b_ and SBP2_9.b_. CC pairs are represented as coloured helices. **h** CD spectra of the proteins SBP1_9.b_ and SBP2_9.b_ and the complex SBP12_9.b_ (cyan, orange and black, respectively) at 20 °C. **i** CD signal at 222 nm of the proteins SBP1_9.b_ and SBP2_9.b_ and the complex SBP12_9.b_ (cyan, orange and black, respectively) during thermal denaturation, the melting temperatures (*T*_m_) are indicated in the panel. **j** SEC-MALS chromatograms and molecular masses for the proteins SBP1_9.b_ and SBP2_9.b_ and the complex SBP12_9.b_. Theoretical Mw(SBP1_9.b_) = 40.0 kDa, Mw(SBP2_9.b_) = 39.7 kDa. UV signal is reported in relative absorbance units (RAU). **k** SAXS ab initio reconstruction superimposed on the molecular model of the SBP12_9.b_ complex displaying the best fit to the experimental data. **l** Experimental SAXS profile of the complex SBP12_9.b_ (black trace) matched well with the theoretical SAXS profile calculated for SBP12_9.b_ model structure (*χ* = 1.4) (orange trace). Error bars in grey represent the standard deviation for each data point in black (mean). Source data are provided as a Source Data file.

To investigate the bottom-up assembly from pre-organised subunits, we set out to construct a pseudo-symmetric heterodimeric CC-based bipyramidal cage. Two 9 CC-forming segment subunits were designed retaining the same topology and building modules used in the single-chain design, with the binding interface composed of three unpaired CC segments in each subunit (Fig. 3a). To increase the strength of the interaction between the complementary subunits, SN peptides at the binding interface were replaced by SH peptides, which possess an increased helical propensity due to introduced salt bridges between residues at *b*, *c* and *f* positions of CC heptad repeats^56,57^ (Supplementary Table 1). To build molecular models of the complex, the CoCoPOD modelling platform was expanded to allow modelling of multichain architectures (see “Methods”).

Initially, two complementary subunits were designed, each with N- and C-termini located at the vertex opposite to the trigonal interface, with all the interfacial CC-forming segments constrained in loops by short linkers (Fig. 3a). The proteins were named SBP1_9.a_ and SBP2_9.a_ according to a nomenclature that includes the name of the polyhedron (split-bipyramid), the number of the subunit and, in the subscript, the number of segments and the permutation chosen. The isolated protein subunits (SDS-PAGE in Supplementary Fig. 2a) exhibited a high content of α-helical secondary structure, which, however, did not change appreciably when the two proteins were mixed in the equimolar ratio (Fig. 3b). Analogously, the CD temperature unfolding experiments showed no significant difference in the stability of the equimolar mixture of the two subunits (SBP12_9.a_) in comparison to the monomers (Fig. 3c). SEC-MALS analysis showed that the two subunits interacted and formed a heterodimeric assembly when mixed in the equimolar ratio (Fig. 3d). Similarly, native PAGE and ITC experiments confirmed the formation of a heterodimer with a 1:1 stoichiometry of binding and a *K*_d_ of 11.6 ± 2.9 nM (Supplementary Figs. 2b, 5b). Solution structure of the complex SBP12_9.a_ was investigated with SAXS. The measured scattering profile fit poorly to a bipyramidal cage model and lacked the maximum at 0.14 Å^−1^ that was observed for the single-chain design (Fig. 3e, f). Moreover, experimentally determined *D*_max_ of 20.0 ± 1.0 nm and *R*_g_ of 5.3 ± 0.2 nm, differed significantly from those observed for BIP18SN (Supplementary Fig. 3a and Supplementary Table 2). Ab initio reconstruction of the molecular envelope from SAXS data suggested that the complex assumed a partially collapsed conformation, lacking an internal cavity (Supplementary Fig. 4b), distinct from the conformation adopted by the single-chain bipyramid BIP18SN (*V*_r_ = 8.2). We attributed the lack of an internal cavity to non-specific interactions at the interface. Further variants of the two subunits, with differences in the interacting interfacial segments, were therefore prepared and tested. These variations included the introduction of segments with decreased helical propensity, different CC building modules and a modified sequential order of CC segments. However, based on SAXS similarity analysis they led in all cases to complexes diverging from the single-chain protein BIP18SN (*V*_r_ values >7.5), indicating incorrect self-assembly (Supplementary Fig. 7).

Taken together, the results suggested that the chosen topology, with constrained unpaired CC segments at the interaction interface, might have been responsible for the collapse of the heterodimeric complex rather than a sequence-specific problem related to the individual subunits. Therefore, we sought to investigate a different circular permutation of the two subunits with two additional designs named SBP1_9.b_ and SBP2_9.b_. In this case, the N- and C-termini were positioned at the binding interface rather than at the opposing vertices (Fig. 3g). In this arrangement, the three CC dimers in each subunit were more constrained than in the SBP12_9.a_ design, while the interfacial CC-forming segments possessed a higher degree of conformational freedom (Supplementary Fig. 8). The subunits SBP1_9.b_ and SBP2_9.b_ were purified separately (Supplementary Fig. 2a) and analysed both alone and in combination. CD analysis revealed a predominantly helical secondary structure for both subunits (*α* = 69 and 73%), which further increased (*α* = 85%) upon mixing the two proteins in an equimolar ratio (Fig. 3h). This increase in the helical content suggested stabilisation of the interfacial helical elements in the case of the heterodimeric mixture, a feature that had not been observed in the SBP12_9.a_ complex. In addition, thermal unfolding experiments monitored by CD spectroscopy revealed that the stability of this two-chain complex (Fig. 3i) was comparable to the single-chain BIP18SN protein (Fig. 1c). SEC-MALS and native PAGE showed that the individual subunits assumed a predominantly monomeric state in solution and associated in a heterodimeric complex only upon mixing (Fig. 3j and Supplementary Fig. 2b). In addition, ITC experiments confirmed a 1:1 binding ratio with a *K*_d_ of 9.4 ± 1.2 nM (Supplementary Fig. 5c). Finally, in contrast to the SBP12_9.a_ complex described above, SAXS profile of the heterodimeric complex SBP12_9.b_ displayed high overall similarity to the scattering curve observed for BIP18SN (*V*_r_ of 1.8) (Fig. 3e, Supplementary Fig. 3b and Supplementary Table 2) with *D*_max_ of 11.8 ± 0.5 nm and *R*_g_ of 4.0 ± 0.1 nm, indicating SBP12_9.b_ assumed a bipyramidal conformation in solution in accordance with the design (Fig. 3k, l and Supplementary Fig. 3a). To elucidate the results obtained from SAXS analysis in the regards of the conformation assumed by the complex SBP12_9.b_, an ensemble of possible conformations was generated using the CoCoPOD software (see “Methods” and Supplementary Software 1) and compared to the experimental SAXS profile (Supplementary Fig. 9). Conformations with an internal cavity displayed a good fit to SAXS data (Fig. 3k, l and Supplementary Fig. 3a), whereas structures with a collapsed cavity did not match the obtained SAXS profile (Supplementary Fig. 9). In addition, ab initio reconstruction of the molecular envelope based on SAXS data confirmed that the complex folded into a bipyramidal shape (Fig. 3k and Supplementary Fig. 4c), as in the single-chain variant BIP18SN.

To investigate the difference in the conformation of the two types of complexes, SBP12_9.a_ and SBP12_9.b_, and understand whether it could be explained by the difference in the pre-organised structures adopted by the individual subunits before binding, SAXS profiles were measured individually for all the differently permuted subunits. This revealed a higher *D*_max_ for the subunits SBP1_9.a_ and SBP2_9.a_ compared to SBP1_9.b_ and SBP2_9.b_ but high similarity in terms of the overall conformation (Supplementary Fig. 8). Due to the structural similarity between the two permuted pairs of subunits, we concluded that topologies that grant the unpaired interfacial CCs a higher degree of conformational freedom—as opposed to being constrained by linkers—facilitate the correct formation of an interface between the individual CCPO subunits.

### Proteolysis-regulated CCPO cage conformational switch

**Fig. 4:**
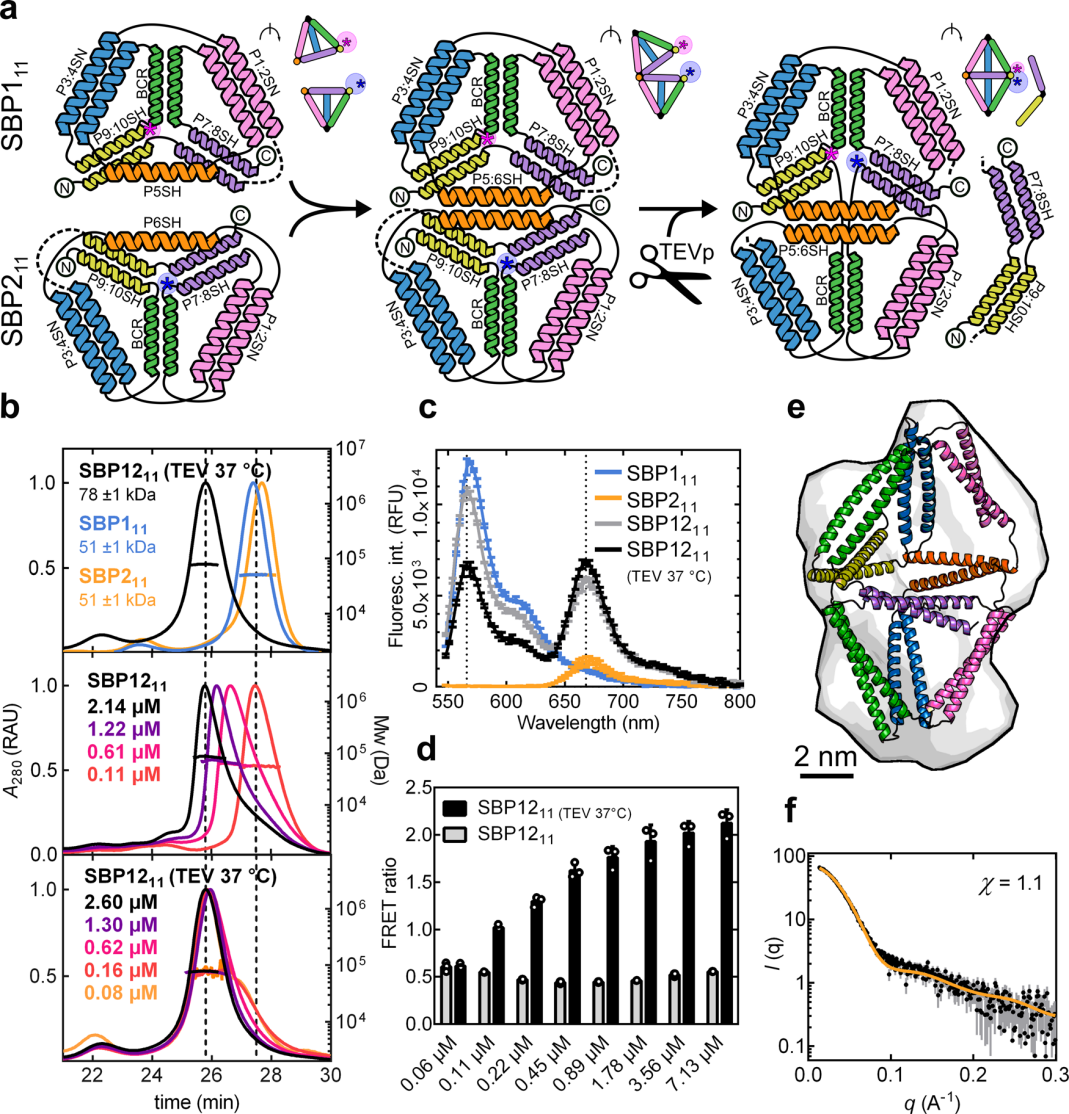
Proteolysis-triggered assembly of the two-chain bipyramidal cage SBP12_11_. **a** Topological scheme of the protein SBP12_11_ before and after TEV proteolytic cleavage; N- and C-termini are indicated with circled letters and the positions of fluorophores are indicated as asterisks. Coloured helices represent different CC-forming segment pairs. The linkers containing the TEV protease cleavage sites are represented with dotted lines. Upper panels show a schematic representation of the protein complex rotated of 60°, indicating the positions of fluorophores as asterisks. **b** SEC-MALS chromatograms: top panel shows individual subunits SBP1_11_ and SBP2_11_ before cleavage (cyan and orange traces, respectively) and the complex SBP12_11_ after treatment with TEV protease (black trace). Central and bottom panels show the complex SBP12_11_ before and after structural rearrangement, eluting in different states at different concentrations. The concentration values correspond to the protein concentrations in the eluted peaks. Molecular weights were calculated from the light scattering signal observed across the main peaks eluting from a size-exclusion column. The theoretical molecular weights of the proteins before cleavage were Mw(SBP1_11_) = 50.8 kDa and Mw(SBP2_11_) = 51.8 kDa and after TEV cleavage Mw(SBP1_11_) = 40.0 kDa and Mw(SBP2_11_) = 41.7 kDa. UV signal is reported in relative absorbance units (RAU). **c** Fluorescence spectra of the two subunits SBP1_11_ and SBP2_11_ labelled with sulfo-cy3 and sulfo-cy5, respectively (cyan and orange traces, respectively) and of the complex SBP12_11_ before and after treatment with TEV protease (grey and black traces, respectively). Error bars represent the standard deviation of three measurements of the same samples (*n* = 3). The fluorescence signal is reported in relative fluorescence units (RFU). **d** The bar graph shows the FRET ratio calculated from measurements at different concentrations of the complex SBP12_11_ before and after treatment with TEV protease (grey and black traces, respectively). Error bars represent the standard deviation of three measurements of the same samples (*n* = 3). **e** SAXS ab initio reconstruction superimposed on the molecular model of the complex SBP12_11_ that best fit the experimental data. **f** SAXS profile of the complex SBP12_11_ after TEV cleavage and removal of the cleaved dipeptide segments (black trace) superimposed on the theoretical SAXS profile of the best-fit model (*χ* = 1.1) (orange trace). Error bars in grey represent the standard deviation for each data point in black (mean). Source data are provided as a Source Data file.

In natural protein architectures, supramolecular self-assembly plays an important role in conformational rearrangement and is associated to activity modulation and allosteric effect^58^. Analogously, in polyhedral CC-based cages, oligomeric assembly could allow the implementation of inter-molecular structural rearrangement mechanisms. To introduce this feature in our CC-based cages, we sought to incorporate a proteolysis-activated structural switch into the heterodimeric bipyramid. Two complementary CC-forming segments were appended to the termini of the subunits SBP1_9.b_ and SBP2_9.b_ (Fig. 4a) to mask the interaction interface. The two subunits formed nearly complete tetrahedral cages comprising 11 CC-forming segments (thus named SBP1_11_ and SBP2_11_) and could interact with each other only through a single complementary edge left unpaired at the binding interface (P5SH and P6SH in SBP1_11_ and SBP2_11_, respectively). Next, a cleavage site for the site-specific Tobacco Etch Virus (TEV) protease was introduced between the 9th and 10th peptide segments to enable trimming off the two terminal CC-forming segments from each subunit. The proteolysis would fully expose the triangular interface for interaction; thereby triggering the structural rearrangement of the dimer into a CCPO bipyramidal cage upon addition of the TEV protease (Fig. 4a).

The two subunits were purified separately (SDS-PAGE in Supplementary Fig. 2a), mixed in equimolar ratio and characterised in the absence and presence of TEV protease. To track changes in quaternary structure, the subunits were labelled with fluorescent dyes in the proximity of the binding interface. Specifically, cysteine residues were positioned between the CC segments P10SH and BCRSH (Cys 201) for SBP1_11_ and between the CC segments BCR and P7SH for SBP2_11_ (Cys 239) (Fig. 4a). The change in Förster resonance energy transfer (FRET) between the two fluorescently labelled subunits was monitored before and after treatment with TEV protease.

SEC-MALS and native PAGE indicated that, due to two non-paired complementary CC segments, the two subunits formed a dimeric complex when mixed together in solution at concentrations above 2 μM already in the absence of TEV protease (Fig. 4b and Supplementary Fig. 2b). However, the FRET efficiency before treatment with TEV protease was comparatively low (FRET ratio = 0.5), suggesting the dyes were not yet in close proximity and the dimer did not assume a bipyramidal shape (Fig. 4c, d). SEC-MALS measurements demonstrated that removing the masking CC segments with TEV protease, followed by incubation at 37 °C, resulted in the displacement of the masking segments and increased binding affinity between the tetrahedral subunits, reflected in dimer formation at lower concentrations (Fig. 4b). Additionally, the heterodimer exhibited a concentration-dependent increase in the FRET ratio (Fig. 4c, d and Supplementary Fig. 10). At higher concentrations, the FRET ratio was approximately four times higher than in the absence of treatment with TEV protease, indicating the two subunits rearranged in closer proximity following the proteolytic event. In the absence of treatment with TEV protease, the incubation at 37 °C promoted an increase in the FRET signal at high concentrations, albeit 50% lower in comparison to the signal obtained for the complex that was incubated with TEV protease (Supplementary Fig. 10). Overall, FRET measured at different concentrations indicated that proteolytic cleavage promoted a structural rearrangement in the heterodimeric bipyramid upon dissociation of the masking CC segments.

The structure of the heterodimeric SBP12_11_ complex after incubation with TEV protease and removal of the two terminal segments was further characterised by SAXS. The scattering profile confirmed the subunits assembled into a bipyramidal conformation, similar to the conformation assumed by the complex SBP12_9.b_, with a *V*_r_ of 5.0, *D*_max_ of 13.5 ± 1.0 nm and a *R*_g_ = 4.2 ± 0.1 nm (Fig. 4d, Supplementary Fig. 3a and Supplementary Table 2). The experimental profile fit a bipyramidal cage model (Fig. 4e, f); moreover, ab initio reconstruction of the molecular envelope based on SAXS data confirmed the presence of an internal cavity and the desired shape (Supplementary Fig. 4d). Taken together, these results showcased the successful implementation of a proteolysis-dependent inter-molecular structural rearrangement mechanism into a CC-based cage.

## Discussion

Modularity is a key element in the design of molecular machines. Accordingly, modular strategies, such as CCPO design, seek to establish the general rules for the assembly of supramolecular architectures and the introduction of dynamic functionalities. Here, we first demonstrated the de novo design of a triangular bipyramid CCPO fold. SAXS and EM analysis confirmed the 18 CC segments protein assembled in the desired conformation. Furthermore, to extend the CCPO design strategy beyond single-chain design, we investigated different strategies for two-chain design, establishing the design rules for developing dynamic multi-chain CC-based polyhedral protein assemblies.

Different approaches were tested for constructing a two-chain trigonal bipyramidal protein fold. The formation of an asymmetrically split complex of two differently sized subunits, interacting via an interface composed of 2 CCs, relied on the interaction of a large subunit composed of 16 CC-forming segments with a smaller 2-CC-forming segment subunit. This approach represents a strategy to enable the incorporation of chemically synthesised or genetically encoded functional elements such as fluorescent reporters, antigens and small molecules linked to short peptides into defined positions of CC-based assemblies.

A protein cage could also be assembled from interacting, pseudo-symmetric, structurally pre-ordered subunits, as demonstrated by the complex SBP12_9.b_. The two subunits formed a bipyramid protein cage only upon mixing and were otherwise monomeric in solution. This approach demonstrated the validity of bottom-up self-assembly for CC-based polyhedral cages utilising pre-organised smaller subunits. Importantly, we found that CC segments placed at the binding interface required a higher degree of conformational freedom to prevent the collapse of the internal cavity and thus allow the cage to adopt the desired conformation. Overall, these findings established a strategy for the use of designed CC-based subunits as building blocks for the assembly of larger oligomeric protein cages, which could in turn increase the complexity of achievable assemblies.

In addition to the two-chain design, we sought to implement a conformational switch into our CC-based protein assembly. Structural modulation and conformational transition are coveted features in protein design. Recently, the design of different de novo proteins responsive to chemical variations has been reported, with examples spanning from conformational change in response to changes in pH^59^, in the presence of divalent cations^60,61^ or via inter-molecular modulation^62^, as well as Zn(II)-responsive CC units^63–65^. Here, we introduced a proteolysis-triggered conformational switch in a heterodimeric CCPO bipyramidal cage. The addition of masking segments that hindered the interaction between tetrahedral subunits and the introduction of a TEV cleavage site for their subsequent removal resulted in a protein complex with tunable interaction properties able to undergo irreversible rearrangement and assemble into a CCPO bipyramid after the proteolytic cleavage. Importantly, responsiveness to a proteolytic cleavage introduces a level of structural modulation controlled directly by adding the appropriate protease or indirectly by adding small molecules that can affect protease activity (e.g., chemically regulated split-proteases)^44^, thus expanding the potential uses of the CCPO design strategy.

In conclusion, we established a framework for the design and better understanding of CC-based polyhedral protein cages, with modularity properties similar yet distinctly different from DNA-based nanostructured designs. By defining the requirements for building oligomeric CC-based protein architectures, we demonstrated the potential of a modular design strategy based on CC building blocks to construct multimeric cages with dynamic properties. In combination with further advances, such as using larger CC sets and implementing topological staples (e.g., protein ligation) and responsive CC elements, this represents a step towards the design of complex CC-based molecular machines.

## Methods

### Preparation of genes and molecular cloning

Cloning passages with recombinant DNA, such as plasmid propagation, mutagenesis and vector transfer, were carried out using the *E. coli* strain DH5-α (F^−^ φ80*lac*ZΔM15 Δ(*lac*ZYA-*arg*F) U169 *rec*A1 *end*A1 *hsd*R17(r_K_^−^, m_K_^+^) *pho*A *sup*E44 λ^−^ *thi*-1 *gyr*A96 *rel*A1) (NEB, MA, USA).

Synthetic genes were purchased from Twist Bioscience (CA, USA) and DNA oligonucleotides used in PCR reactions were purchased from IDT (IA, USA). Genes coding for the proteins of interest were cloned in the expression vector pET41a(+) (Genscript, NJ, USA) between the restriction sites NdeI and XhoI, and reading frames were optimised for *E. coli* codon usage using a software property of IDT (IA, USA).

Gibson assembly^66^ was used in order to introduce, substitute or delete DNA segments in the genes. Amplification of DNA fragments and vectors (primers in Supplementary Table 3) was performed with KAPAHiFi™ HotStart DNA polymerase (Roche, Switzerland) or Phusion^®^ HotStart DNA polymerase (NEB, MA, USA) in PCR reactions performed according to manufacturer instructions. Gibson assembly was performed with a mixture of the enzymes Taq Ligase (NEB, MA, USA), Phusion^®^ Polymerase (NEB, MA USA) and T5 exonuclease (NEB, MA, USA) in reaction buffer, as previously described^66^. The mixture was incubated for 1 h at 50 °C before transformation in competent *E. coli* cells. For the constructs SBP_16_ and SBP_2_, restriction of PCR products and plasmid was performed with the enzymes NdeI and XhoI (NEB, MA, USA) as indicated by the manufacturer, followed by ligation with T4 ligase (NEB, MA, USA) and transformation. DNA extraction and purification from agarose gel were performed with Spin Miniprep Kit (QIAGEN, Germany).

Plasmid transformation was performed via heat shock with competent *E. coli* cells prepared according to the manufacturer’s indication. Single clones were grown in presence of the antibiotic Kanamycin (Goldbio, MO, USA), 50 µg/ml were added to Lysogeny broth (LB) media.

### Protein production

For protein production we transformed expression vectors containing the protein of interest in *E. coli* strain NiCO21(DE3) (*can::CBD fhuA2 [lon] ompT gal (λ DE3) [dcm] arnA::CBD slyD::CBD glmS6Ala ∆hsdS λ DE3* = *λ sBamHIo ∆EcoRI-B int::(lacI::PlacUV5::T7 gene1) i21 ∆nin5*) (NEB, MA, USA).

Protein overexpression in *E. coli* was obtained by fermentation in Erlenmeyer flasks. Stock cultures were inoculated in 100 ml LB media supplemented with antibiotics (Kanamycin 50 µg/ml) and incubated at 37 °C, 160 RPM overnight. Precultures were diluted to 0.1 OD in larger (5 l) Erlenmeyer flasks filled with 1 l or 1.5 l of LB media supplemented with antibiotics (for a total volume from 2 to 6 l) and left growing at 37 °C before reaching stationary phase. At OD values between 0.6 and 0.9 the cultures were induced with 1 mM IPTG (Goldbio, MO, USA) and grown for four hours in agitation (160 RPM) at 30 °C. Afterwards, the bacteria were harvested via centrifugation and frozen.

Cellular pellets were resuspended in 8.5 ml of lysis buffer (50 mM Tris-HCl at pH 8.0, 150 mM NaCl, 10 mM imidazole, 0.5 mg/ml Lysozyme (Millex Sigma-Aldrich, MO, USA), 18 U/ml Benzonase (Merck, Germany), 1 mM MgCl_2_, 2 µl/ml CPI (Protease Inhibitor Cocktails) (Millex Sigma-Aldrich, MO, USA) per litre of culture. Cell lysis was completed either by ultrasonication or by thermal lysis. Ultrasonication was conducted with a Vibra-cell VCX (Sonics, CT, USA) on ice for maximum four cycles of 1 min of total pulse time, at intervals of 1 s pulse and 3 s pause (55% amplitude). In the case of thermal lysis, the cellular pellets were resuspended in 20 ml of lysis buffer per litre of culture and incubated for 15 min in boiling water, cooled in ice and supplemented with an additional 0.06 µl/ml of Benzonase (250 U/ml) (Merck, Germany) prior centrifugation.

The cellular lysates were centrifuged at 16,000 × *g* (4 °C) for 20 min. The soluble fraction was then filtered through 0.45-μm filter units (Sartorius stedim, Germany) and applied to further purification passages.

### Protein chromatography

A standard isolation protocol was composed of two chromatography steps: affinity (Ni-NTA) and size-exclusion chromatography (SEC); however, in some cases (proteins: BIP18SN, SBP_16_, SBP_15_, SBP1_9.a_, SBP2_9.a_ SBP1_9.b,_ SBP2_9.b_), the protocol required the addition of ion-exchange chromatography (IEX) or a Strep-tag affinity passage (only for SBP2_11_) between Ni-NTA and SEC passage. The proteins SBP1_11_ and SBP2_11_ contained a cysteine residue (used for maleimide labelling) and were therefore isolated in presence of 1 mM TCEP.

Soluble fractions of bacterial lysates after filtration were flushed in 5 ml of Ni-NTA resin (Goldbio, MO, USA) previously equilibrated with buffer A (50 mM Tris-HCl pH 8.0, 150 mM NaCl, 10 mM imidazole) in plastic columns. After washing extensively with buffer A (~400 ml) and buffer B (50 mM Tris-HCl pH 8.0, 150 mM NaCl, 20 mM imidazole) (~500 ml) the bound fraction was eluted with buffer C (50 mM Tris-HCl pH 8.0, 150 mM NaCl, 250 mM imidazole).

For size-exclusion chromatography (SEC), we used HiLoad Superdex™ 200 resin (GE Healthcare, IL, USA), packed in a 26/600 XK column (GE Healthcare, IL, USA) and a HiLoad Superdex™ 75 resin (GE Healthcare, IL, USA) (for SBP_2_ and SBP_3_), packed in a 10/600 XK column (GE Healthcare, IL, USA) equilibrated with filtered and degassed SEC buffer (20 mM Tris-HCl pH 7.5, 150 mM NaCl, 10% v/v glycerol). Samples eluted from Ni-NTA (or from IEX) were concentrated with centrifugal filters (3 K, 10 K or 30 K) (Amicon-ultra, Millex Sigma-Aldrich, MO, USA), and after filtration in 0.22-µm syringe filters (Millex Sigma-Aldrich, MO, USA) were injected into the column. The chromatography was run with an AKTA™ pure FPLC system (GE Healthcare, IL, USA) in SEC buffer with a linear flow rate of 2.6 ml/min or 1 ml/min for Superdex™ 200 and Superdex™ 75, respectively, and the eluted protein fractions were collected separately.

For ion-exchange chromatography (IEX), 10 ml of the anionic exchanger DEAE-Sepharose™ resin (GE Healthcare, IL USA) were packed in a 16/100 XK column (GE Healthcare, IL, USA) and conditioned in filtered and degassed IEX buffer (50 mM Tris-HCl pH 8.0, 150 mM NaCl). Samples eluted from Ni-NTA were filtered with 0.22 µm syringe filters (Millex Sigma-Aldrich, MO, USA) and loaded into the column. After extensive washing with IEX buffer, we established a linear gradient against IEX buffer B (50 mM Tris-HCl pH 8.0, 2 M NaCl), NaCl reached a final concentration of 550 mM in 30 or 50 ml at 1 ml/min, the eluted proteins were collected in separated fractions.

Strep-tag affinity, needed only for the protein SBP2_11_, was performed according to manufacturer instructions with 4 StrepTrap™ 5 ml columns (GE Healthcare, IL USA) connected in series and conditioned with IEX buffer supplemented with 1 mM EDTA and 1 mM TCEP. After binding and washing the protein was eluted with 2.5 mM d-Desthiobiotin (Millex Sigma-Aldrich, MO, USA) in IEX buffer.

All the heterodimeric protein complexes described in the article were obtained by combining the purified subunits in equimolar ratio at low concentration (below 1 mg/ml) to avoid non-specific binding and aggregation. The mixture was then concentrated and purified via an additional SEC passage. The heterodimeric complexes were collected after separation and further concentrated for additional characterisation.

### TEV protease cleavage

The TEV protease was produced following the above-described protocol, encompassing ultrasonication, Ni-NTA and SEC chromatography.

TEV protease was used for performing the cleavage of the 8xHis-tag in the case of the monomeric proteins SBP1_9.a_, SBP2_9.a_, SBP1_9.b_ and SBP2_9.b_ before mixing the two subunits, whereas cleavage of the proteins SBP1_11_ and SBP2_11_ was initiated only after mixing them in equimolar ratio.

Proteins subjected to controlled proteolysis were incubated overnight at 4 °C with the addition of 50 µg of TEV protease per mg of target protein (~50–200 molar excesses of target protein). Subsequently, in order to promote dissociation from the cleaved products (consisting of only affinity tags or tagged 2-helix-long segments as in the case of SBP1_11_ and SBP2_11_) the sample was incubated at 37 °C for 15 min and the mixture was flown through 2.5 ml of Ni-NTA resin (Goldbio, MO, USA) previously conditioned in IEX buffer; the eluted sample was then collected for further analysis.

### Protein electrophoresis

Samples were analysed by SDS-PAGE^67^ in a Bio-rad (CA, USA) mini-PROTEAN™ apparatus in 12% discontinuous polyacrylamide gels containing sodium dodecyl sulphate (SDS). The molecular weight was calculated with a pre-stained molecular ruler (Thermo Fisher Scientific, MA, USA). Native PAGE^68^ was run in a Bio-rad (CA, USA) mini-PROTEAN™ apparatus in 10% discontinuous polyacrylamide gels at a voltage of maximum 120 V at 4 °C, the samples were loaded next to NativeMark™ standards (Thermo Fisher Scientific, MA, USA). All the gels were stained with InstantBlue™ (Millex Sigma-Aldrich, MO, USA). Pictures of uncropped gels are included in the Source Data file.

### Circular dichroism

A Chirascan CD spectrometer equipped with a Peltier temperature controller (Applied Photophysics, UK) was used to record the CD spectra in far-UV (200–280 nm) of protein samples with a concentration ranging between 0.3 mg/ml and 0.5 mg/ml in a 1-mm cuvette (Hellma, Germany) at 20 °C using 1-nm steps, 1-nm bandwidth, and 1 s sampling. Thermal denaturation experiments were conducted with a temperature gradient of 1 °C per minute for heating the sample from 5 °C to 92 °C followed by rapid temperature quenching. CD signal was measured at 222 nm. Experimental curves were fitted with a two-state or three-state equilibrium model described by Drobnak et al.^69^. The helical content of the proteins was calculated according to the following equation:

1 $$\alpha (\% ) = MRE_{222}/(MRE_{222}^H \times (1 - 2.57/n))$$

where *n* is the length of the amino acid sequence, *MRE*_*222*_ average mean residue ellipticity at 222 nm and $MRE_{222}^H$ is the theoretical mean residue ellipticity of an infinitely long helix (−39,500 deg cm^2^ dmol^−1^)^70^.

### Size-exclusion chromatography coupled to multi-angle light scattering

SEC-MALS measurements were performed with an HPLC system (Waters, MA, USA), coupled to a UV detector, a Dawn8+ multiple-angle light scattering detector (Wyatt, CA, USA) and a refractive index detector RI500 (Shodex, Japan). Protein samples were filtered through Durapore 0.1-μm centrifuge filters (Merck Millipore, MA, USA) and injected onto a Superdex™ 200 increase 10/300 column (GE Healthcare, IL, USA) previously equilibrated with SEC buffer B (20 mM Tris-HCl pH 7.5, 150 mM NaCl). Analysis of the peaks of interest was performed using Astra 7.0 software (Wyatt, CA, USA).

### Computational modelling

Molecular models of designed single-chain and oligomeric CCPO bipyramid cages were built using the CoCoPOD software^46^, the updated source code of the software is provided with this paper (Supplementary Software 1). Briefly, the amino acid sequence is designed by selecting an appropriate CC peptide for each position in the polypeptide chain. Next, based on the amino acid sequence a straight α-helix is generated for each polypeptide chain. The initial cage structure is then generated using a multi-step molecular dynamics procedure. During the simulation, each peptide segment is described as a rigid body. In each step, an additional pair of harmonic restraints is added to the force field describing CC pairing between conjugate peptides. The initial model is then refined using homology modelling, employing crystal structures or CC dimer models generated using ISAMBARD software^71^ as a template. To account for flexibility in the structure of CCPO cages, the model building cycle is repeated 30–60 times to generate an ensemble of possible conformations.

### Small-angle X-ray scattering

Scattering curves were measured at P12 beamline of PETRA III – DESY (Hamburg, Germany)^72^ and SIBYLS beamline at ALS (Berkeley, CA, USA)^73^. SAXS experiments performed at PETRA III were conducted at X-ray wavelength of 1.24 Å with the Pilatus 6 M detector positioned at 3 m from the sample. The resulting range of the scattering vector was 0.028–7.3 nm^−1^. Batch measurements were performed with a robotic sample changer in flow-through mode, to avoid radiation damage. For each sample (40 μL), data were collected over 20 exposures each of 0.05 s. Frames not displaying any radiation damage were then automatically averaged and integrated into the SASFLOW pipeline^74^. Before and after, each sample buffer scattering was collected for background subtraction. To assess concentration effects, a dilution series consisting of four concentrations in the range of 8 mg/ml to 1 mg/ml was measured for the single-chain protein BIP18SN. SEC-SAXS was performed with a Superdex™ 200 increase 10/300 column (GE Healthcare, IL USA) in SEC buffer C (20 mM Tris-HCl pH 7.5, 150 mM NaCl, 3% v/v glycerol). The mobile phase was flown into the column at a flow of 0.5 ml/min or 0.6 ml/min. In total, 3000–3600 scattering frames were collected with an exposure time of 0.995 s. SAXS experiments at SIBYLS beamline were performed at X-ray wavelength of 1.03 Å with sample-detector distance (Pilatus3 2 M pixel array detector) of 1.5 m. The scattering vector ranged from 0.13 to 5 nm^−1^. Each frame resulted from 3 s exposures. Frames belonging to the peak of interest were carefully averaged. The contribution of the mobile phase to scattering was eliminated by subtracting averaged frames corresponding to the buffer. Analysis of scattering curves and ab initio modelling was performed using the ATSAS suite^75^. Internal cavities of ab initio models were evaluated with PyMOL Molecular Graphics System. Theoretical SAXS profiles were calculated from molecular models and compared to experimental data using Pepsi-SAXS^76^. The agreement between theoretical and experimental curves was evaluated using the *χ* metric, with low values signalling a good fit.

Experimental scattering profiles were compared using the volatility ratio (*V*_R_). *V*_R_ was calculated by taking the ratio of two scattering profiles in the scattering vector range of 0.15–1.5 nm^−1^. The ratio was binned at frequency *q* = π/d, assuming *d* = 40 nm and the average ratio was calculated for each bin. Volatility ratio was then calculated as:

2 $$V_R = \frac{1}{N}\sum_{i = 1}^N {\left| {\frac{{R\left( i \right) - R(i + 1)}}{{\left( {R\left( i \right) + R\left( {i + 1} \right)} \right)/2}}} \right| \times 100}$$

where *R(i)* is the ratio for bin *i* and N the number of bins.

### Negative-stain electron microscopy

The purified protein sample BIP18SN was diluted in SEC buffer to a final concentration of 20 μg/mL and applied to a glow-discharged carbon-coated copper grid. Afterwards, the grid was briefly washed with distilled water, stained negatively with 2% (w/v) uranyl acetate and observed using a JEOL-1230 functioning at 100 kV. Single particles were imaged automatically using a TVIPS F416 CMOS at a final magnification of 54,926. The image processing was carried out through the Scipion platform (http://scipion.cnb.csic.es)^77^. Around 50,000 particles were extracted from 150 micrographs and classified in 2D with the software Xmipp^78^. Approximately 20,000 particles were used for the refinement passages. The software UCSF Chimera^79^ was used to fit the molecular model of BIP18SN that best matched the SAXS profile into the 3D EM reconstruction via a global search of the best orientation.

### Isothermal titration calorimetry

An isothermal titration calorimeter MicroCal VP-ITC (Malvern Panalytical, UK) was used for the experiments. An excess of the titrant species (volumes of 300 ml) at a concentration of 7–15 µM and 15 µM was loaded in a stirring syringe, and a volume of 1.4 ml of analyte solution at 0.8–1.3 µM was loaded in the isothermal cell. After initial equilibration (6000 s), the analyte was titrated with 27–30 additions of 5–10 µl of titrant in the syringe at intervals of 1600 s. The first injection always consisted of 2 µl of the titrant. In the case of SBP2_9.b_, the volume of 2nd to 7th injection was of 5 µl. The heat effects were obtained by integration and fitted to a 1:1 dissociation model with software developed by Drobnak et al.^69^.

### Protein labelling and fluorescence measurements

After isolation in presence of 1 mM TCEP, the proteins SBP1_11_ and SBP2_11_ were mixed individually with 10× molar excesses of the dyes maleimide-sulfo-Cy3 and maleimide-sulfo-Cy5, respectively, and incubated overnight at 4 °C. The dyes were purchased from Lumiprobe (MD, USA), kept at −20 °C and dissolved in DMSO prior use. Following the reaction, the excess of dye was removed via desalting using PD-10 desalting columns (GE Healthcare, IL, USA). The ratio Protein/Dye was calculated by measuring the UV–visible spectra of the eluted products. The fluorescence of conjugated proteins was measured in a final volume of 100–50 µl with a multi-plate fluorescence reader Synergy Mx (BioTeK, VT, USA). The emission spectra of individual proteins SBP1_11_ and SBP2_11_ and their equimolar mixture were recorded from 548 nm to 800 nm (bandpass 9 nm) upon excitation at 528 nm (bandpass 9 nm). We incubated the equimolar combination of SBP1_11_ and SBP2_11_ overnight in the presence or absence of TEV protease (50 molar excesses of target protein). Afterwards, in order to favour the dissociation of the 2-CC-long-segment, all the samples were incubated at 37 °C for 15 min and cooled down at room temperature. The emission spectra were measured again for all the samples. The FRET ratio was calculated as the emission of the acceptor (SBP2_11-cy5_) at 668 nm over emission of the donor (SBP1_11-cy3_) at 566 nm according to the following equation:

3 $$FRET\;ratio = \frac{{F(A)}}{{F(D)}}$$

where *F(A)* stands for the emission of acceptor and *F(D)* for the emission of the donor at different concentrations of the equimolar mixture. The measurements were repeated three times (*n* = 3) for each combination of donor and acceptor and averaged.

### Software and statistics

Graphs were prepared with Gnuplot 5.0 (http://www.gnuplot.info/), Matplotlib 2.0.1 (https://matplotlib.org/) and GraphPad Prism (https://www.graphpad.com/). Results from SEC-MALS and SAXS were analysed as described in “Methods”. SAXS at EMBL-DESY data was acquired and initially analysed with the SASFLOW pipeline^74^. Figures were generated with Inkscape (https://inkscape.org/). Images of molecular models were created using UCSF Chimera (https://www.cgl.ucsf.edu/chimera/) and the PyMOL Molecular Graphics System, Version 2.3 Schrödinger, LLC (https://pymol.org/2/). The amino acid contact map was generated using CMView^80^ (http://www.bioinformatics.org/cmview). ITC data were collected with the software VPViewer 1.4.12 (Malvern Panalytical, UK). Negative-stain EM image processing was performed using the Scipion platform (http://scipion.cnb.csic.es/). Fluorescence spectra and intensities were recorders with the software Gen5 (BioTek, VT, USA). The updated source code of the CoCoPOD software is provided with this article (Supplementary Software 1).

### Reporting summary

Further information on research design is available in the Nature Research Reporting Summary linked to this article.

## Supplementary information

Supplementary Information

Description of Additional Supplementary Files

Supplementary Software 1

Reporting Summary

## Data Availability

EM data for BIP18SN have been deposited into EMDB (www.ebi.ac.uk/pdbe/emdb) with accession code EMD-11831. SAXS scattering data has been deposited into SASBDB (www.sasbdb.org) with accession codes: SASDJU5 for BIP18SN and SASDJV5 for the complex SBP12_9.b_. Source data are provided with this paper.

## Source data

Source Data

## Code availability

The source code of the software used for evaluating CCPO cages’ topology and for designing molecular models is provided with this paper (Supplementary Software 1). The software is distributed under the MIT license.
