# Author Correction: Self-assembly and regulation of protein cages from pre-organised coiled-coil modules

**DOI:** 10.1038/s41467-021-21969-9

**Published:** 2021-03-16

**Authors:** Fabio Lapenta, Jana Aupič, Marco Vezzoli, Žiga Strmšek, Stefano Da Vela, Dmitri I. Svergun, José María Carazo, Roberto Melero, Roman Jerala

**Affiliations:** 1 Department of Synthetic Biology and Immunology, National Institute of Chemistry, Ljubljana, Slovenia; 2 EN-FIST Centre of Excellence, Ljubljana, Slovenia; 3 Department of Chemistry, Life Sciences and Environmental Sustainability, University of Parma, Parma, Italy; 4 EMBL c/o DESY, 2607 Hamburg, Germany; 5 Centro Nacional de Biotecnología (CNB-CSIC), Madrid, Spain

**Keywords:** Synthetic biology, Nanostructures, Protein design, SAXS

Correction to: *Nature Communications* 10.1038/s41467-021-21184-6, published online 11 February 2021.

The original version of this Article contained an error in Fig. 3a, in which the topological scheme representing the protein SBP1_9.a_ had one linker misplaced and one missing. This has been corrected in both the PDF and HTML versions of the Article.

