# Supplementary material for: Self-assembly and regulation of protein cages from pre-organised coiled-coil modules
Source: Nat Commun. 2021 Feb 11;12:939. doi: 10.1038/s41467-021-21184-6 (PMC7878516; doi:10.1038/s41467-021-21184-6)
Supplement: Supplementary file 1 — Supplementary Information [file 41467_2021_21184_MOESM1_ESM.pdf]

Supplementary Information

Self-assembly and regulation of protein cages from pre-organised coiled-coil modules

Fabio Lapenta[1,2], Jana Aupič[1], Marco Vezzoli[3], Žiga Strmšek[1], Stefano Da Vela[4], Dmitri I. Svergun[4], José María Carazo[5], Roberto Melero[5] and Roman Jerala[1,2,*]

[1] Department of Synthetic Biology and Immunology, National Institute of Chemistry, Hajdrihova 19, SI-1000 Ljubljana, Slovenia

[2] EN-FIST Centre of Excellence, Trg OF 13, SI-1000 Ljubljana, Slovenia

[3] Department of Chemistry, Life Sciences and Environmental Sustainability, University of Parma, Parco Area delle Scienze 11/a 43124 Parma, Italy

[4] EMBL c/o DESY, Notkestr. 85, 22607 Hamburg, Germany

[5] Centro Nacional de Biotecnología (CNB-CSIC), Madrid, Spain.

* Correspondence to: roman.jerala@ki.si

This document includes:

- Supplementary Figures 1 to 10
- Supplementary Tables 1 to 3
- List of protein sequences
- List of DNA sequences

Supplementary Figures

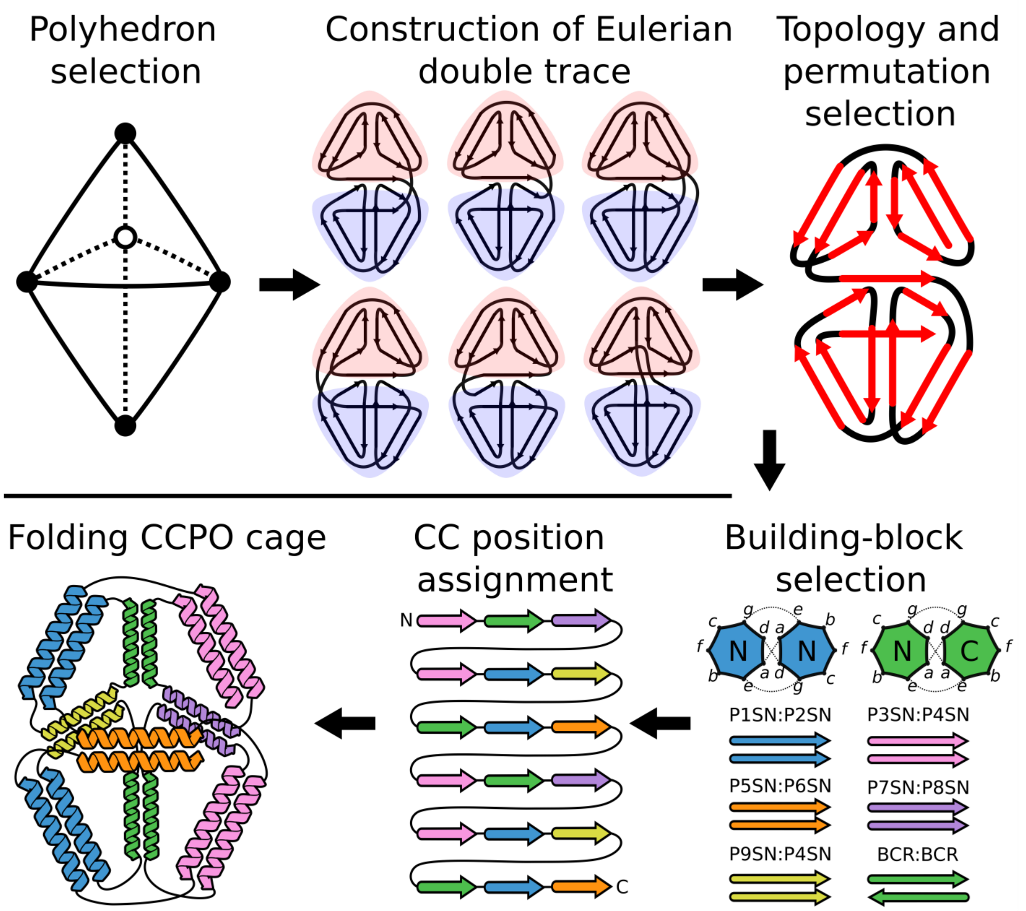

Supplementary Figure 1: Design process for the modular trigonal bipyramid cage BIP18SN. From left to right: i) the selection of a polyhedral shape was followed by ii) tracing a double Eulerian trail for different topological solution of two pseudo-symmetric tetrahedral halves. iii) The selection of the beginning and end of the linear chain was based on the selection of the lowest topological contact order (TCO) between the complementary edges of the polyhedron. iv) The CC building blocks were selected from a set of orthogonal CC dimers. v) The building blocks were assigned to different positions, prioritising stronger pairs at the termini and using the same pairs in each half as indicated by the colour. vi) Molecular models of the protein were built and used to fit experimental data.

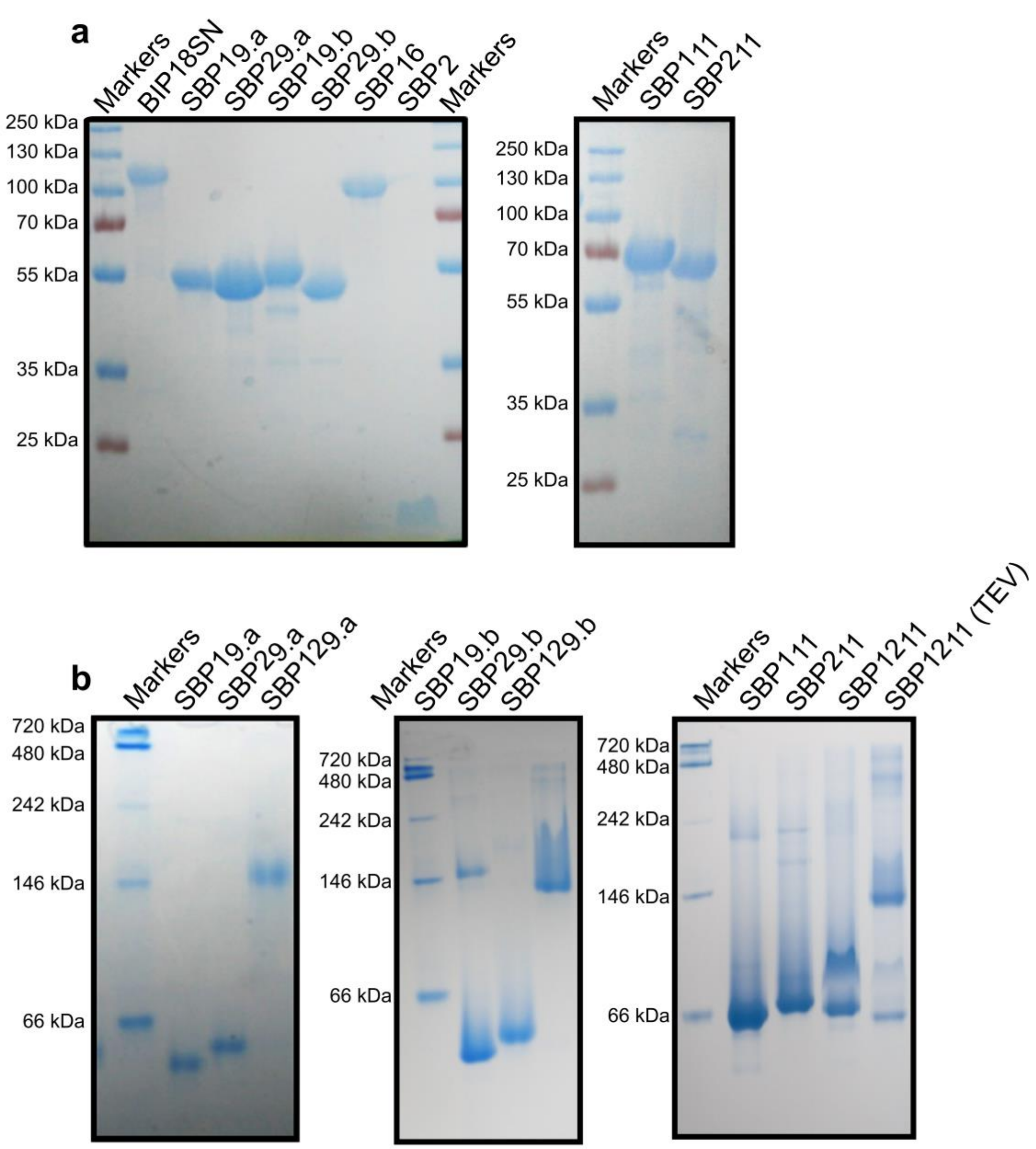

Supplementary Figure 2: Electrophoretic analysis of purified proteins. a, SDS-PAGE of purified proteins loaded next to molecular weight markers. Markers from bottom to top: 25 kDa, 35 kDa, 55 kDa, 70 kDa, 100 kDa, 130 kDa, 250 kDa. **b**, Native-PAGE of monomeric proteins and protein complexes before and after treatment with TEV protease at 37 °C loaded next to molecular weight markers. Markers from bottom to top: 66 kDa, 146 kDa, 242 kDa, 480 kDa, 720 kDa. The gels are representative of at least two repetitions of the same experiments. Source data are provided as a Source Data file.

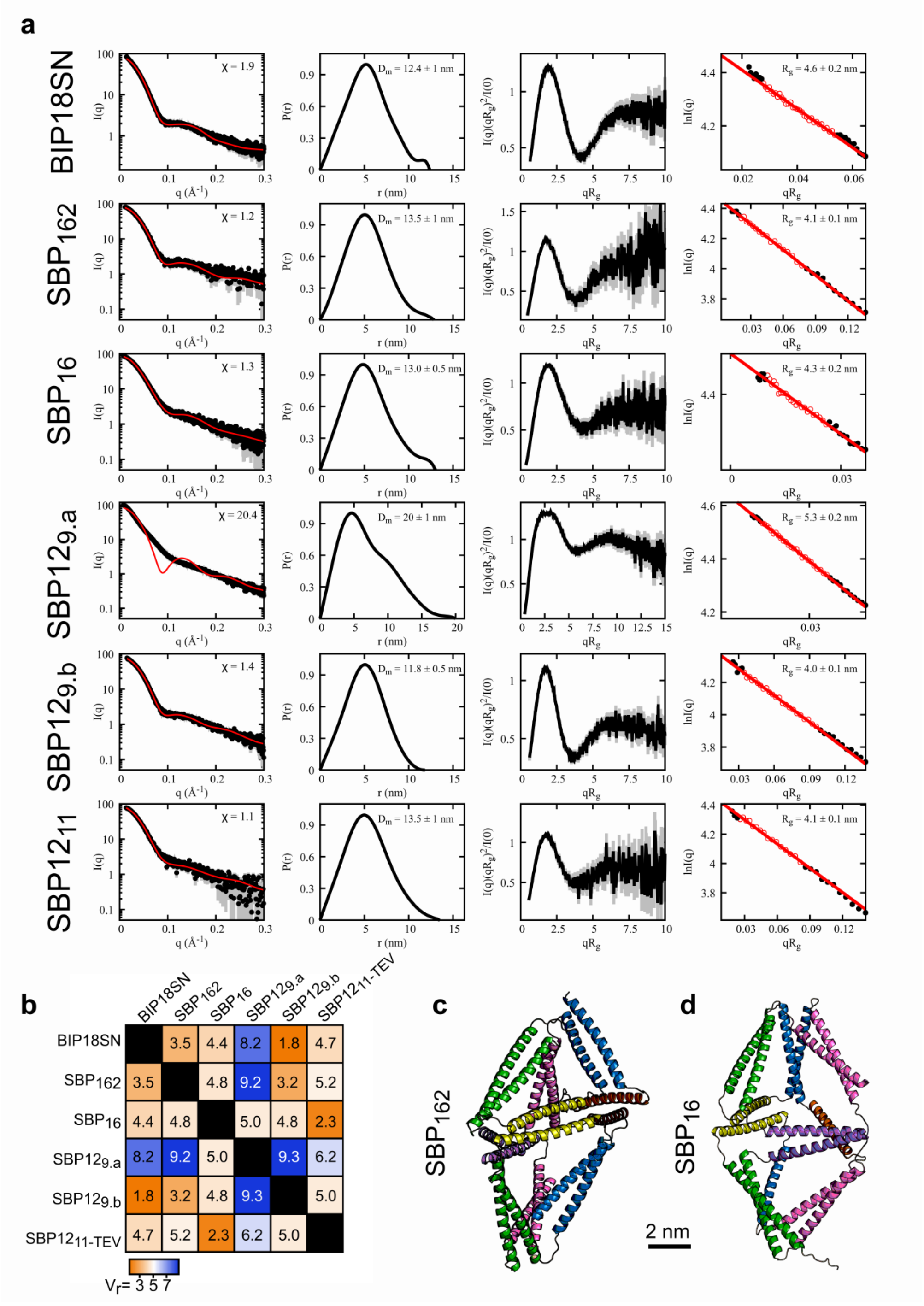

Supplementary Figure 3: SAXS analysis of CCPO bipyramidal proteins. a, SAXS analysis of main CCPO bipyramidal proteins. From left to right: SAXS scattering curve (black trace for experimental SAXS data and red trace for theoretical SAXS curve calculated from molecular model of the cage structure), pair distribution function; normalised Kratky plot and Guinier plot. Error bars in grey represent the standard deviation for each data point in black (mean). **b**, The bottom panel contains a similarity matrix with the volatility ratio for

each protein calculated as described in the Methods section. **c**, Molecular model of SBP_{162} used to fit experimental SAXS curve. **d**, Molecular model of SBP_{16} used to fit SAXS results. Source data are provided as a Source Data file.

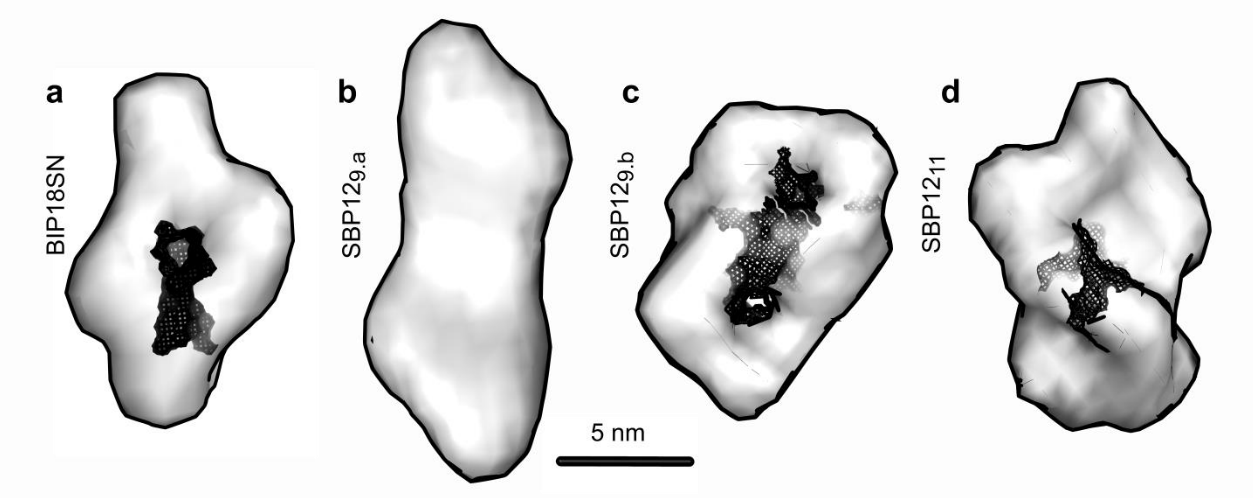

Supplementary Figure 4: *Ab initio* reconstruction of the molecular shape based on SAXS data. *Ab initio* SAXS reconstruction based on the pair distance distribution function for **a**, BIP18SN; **b**, $SBP12_{9.a}$; **c**, $SBP12_{9.b}$**,** **d**, $SBP12_{11}$. The cavities are visible as black mesh surfaces.

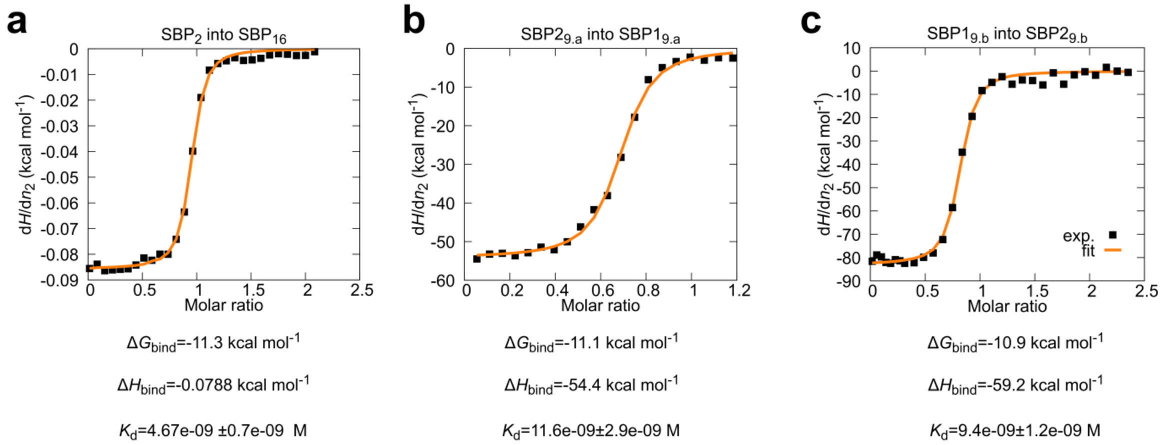

Supplementary Figure 5: Isothermal titration calorimetry of interactions between the CCPO modules. ITC experiments were used to determine the K_d for the formation of the heterodimeric protein complexes **a**, SBP$_{162}$, **b**, SBP12$_{9.a}$ **c**, SBP12$_{9.b}$. In each case, the experimental data was described with a 1:1 binding model to obtain the free energy and the enthalpy of binding. Source data are provided as a Source Data file.

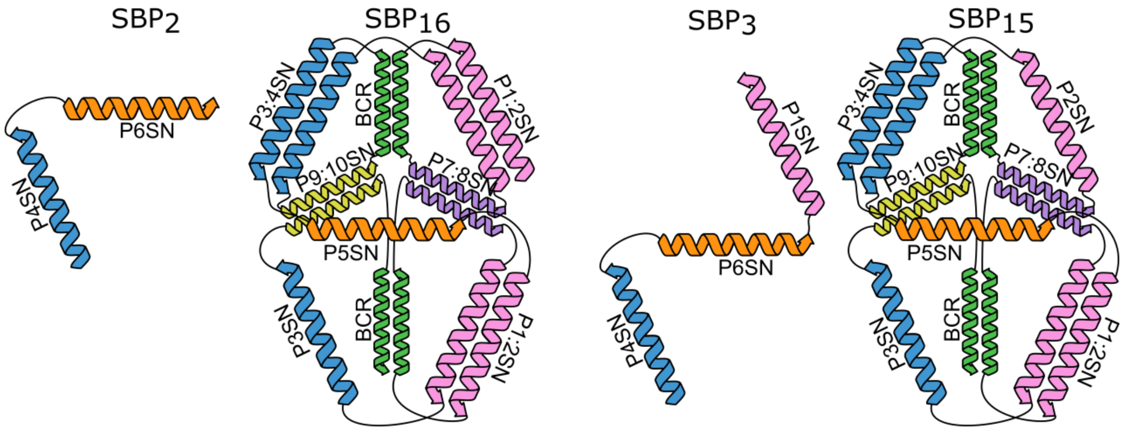

Supplementary Figure 6: Topological schemes of the asymmetric assembly of SBP heterodimers. The schemes represent different protein designs used for the asymmetric reconstruction of the heterodimeric bipyramid. While the complex SBP$_2$:SBP$_{16}$ was fully characterised, the protein complex deriving from SBP$_{15}$ and SBP$_3$ was not characterised due to the low solubility of the subunit SBP$_{15}$.

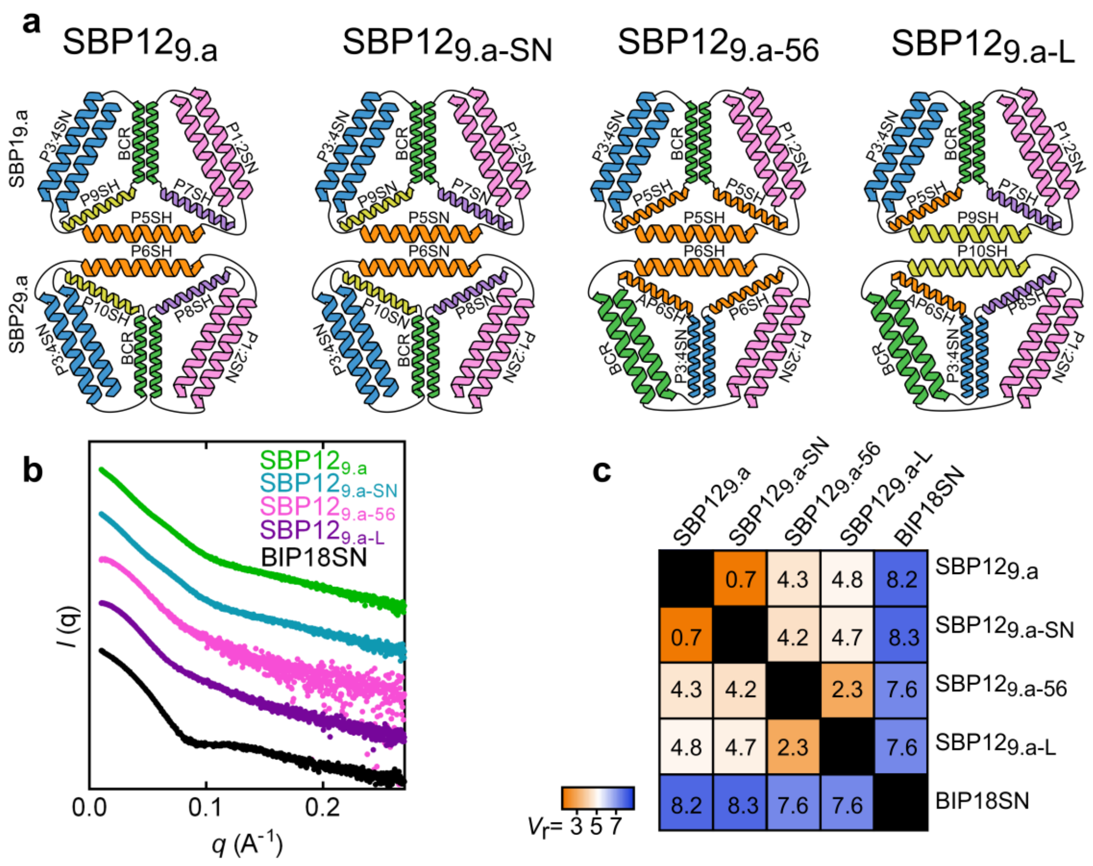

Supplementary Figure 7: SAXS analysis of alternative SBP12$_{9.a}$ protein complexes. a, Topological schemes of different variants of the protein complex SBP12$_{9.a}$. Coiled-coil pairs are represented as coloured helices. The complex SBP12$_{9.a}$ is described in the main text. The complex SBP12$_{9.a-SN}$ is analogous to the former; however, CC segments with decreased helicity have been positioned at the interface (SN instead of SH). The complex SBP12$_{9.a-56}$ has been designed employing a repetition the same CC segments P5SH:P6SH at interfacial positions. The complex SBP12$_{9.a-L}$ presents only a different arrangement of CC segments compared to the complex SBP12$_{9.a}$. **b**, SAXS scattering curves of (from top to bottom) SBP12$_{9.a}$, SBP12$_{9.a-SN}$, SBP12$_{9.a-56}$, SBP12$_{9.a-L}$ and BIP18SN. **c**, V_r matrix showing a comparison between experimental SAXS profiles obtained for different variants of the complex SBP12$_{9.a}$ and the single-chain protein BIP18SN. Source data are provided as a Source Data file.

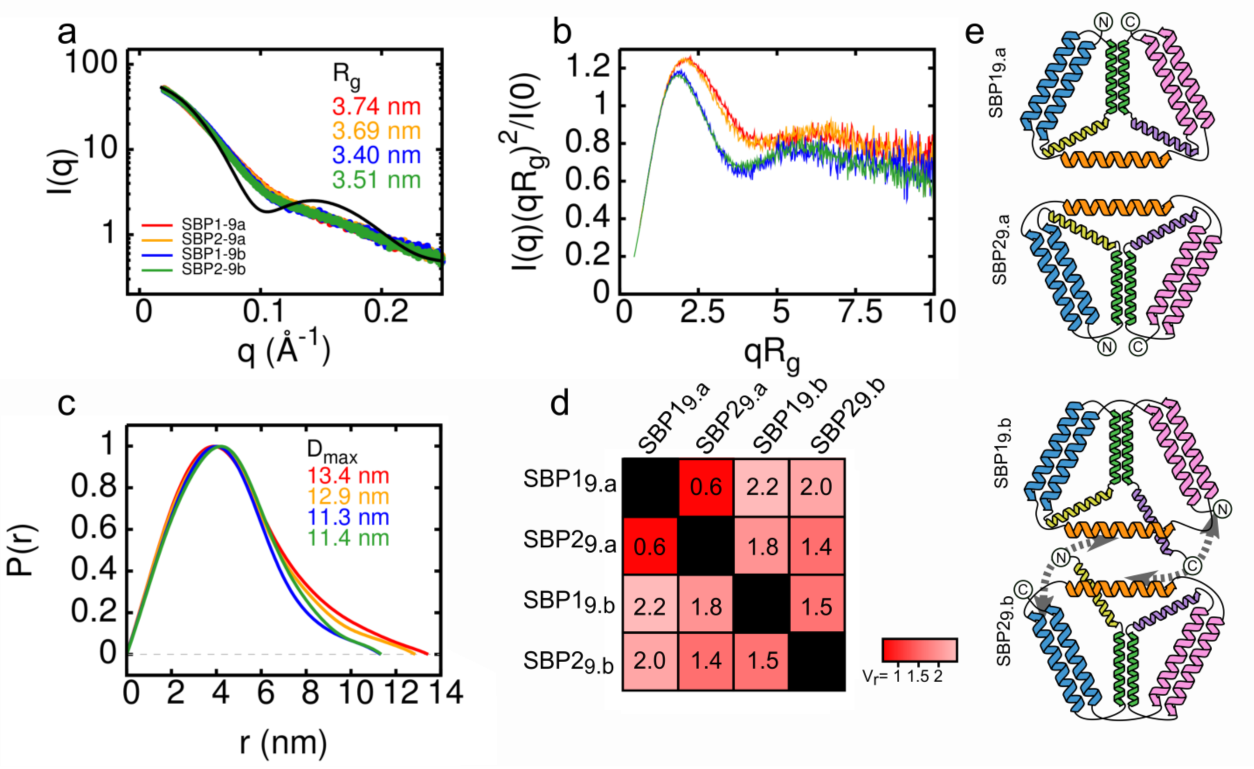

Supplementary Figure 8: SAXS analysis of individual $SBP_{9.a}$, $SBP_{9.b}$ subunits. a, SAXS profiles of individual split-bipyramid subunits with R_g (black, red, yellow, blue and green traces for a theoretical bipyramid model, $SBP1_{9.a}$, $SBP2_{9.a}$, $SBP1_{9.b}$, $SBP2_{9.b}$ respectively). **b**, Normalised Kratky plot of individual split-bipyramid subunits. **c**, SAXS pair distance distribution function for individual split-bipyramid subunits. **d**, V_r matrix showing the similarity of SAXS profiles measured for individual split-bipyramid subunits. **e**, Topological schemes of the subunits where coiled-coil pairs are represented as coloured helices. N- and C-termini are indicated with circled letters. Dashed arrows indicate the higher degree of freedom for the termini of the subunits $SBP1_{9.b}$ and $SBP2_{9.b}$ compared to $SBP1_{9.a}$ and $SBP2_{9.a}$. Source data are provided as a Source Data file.

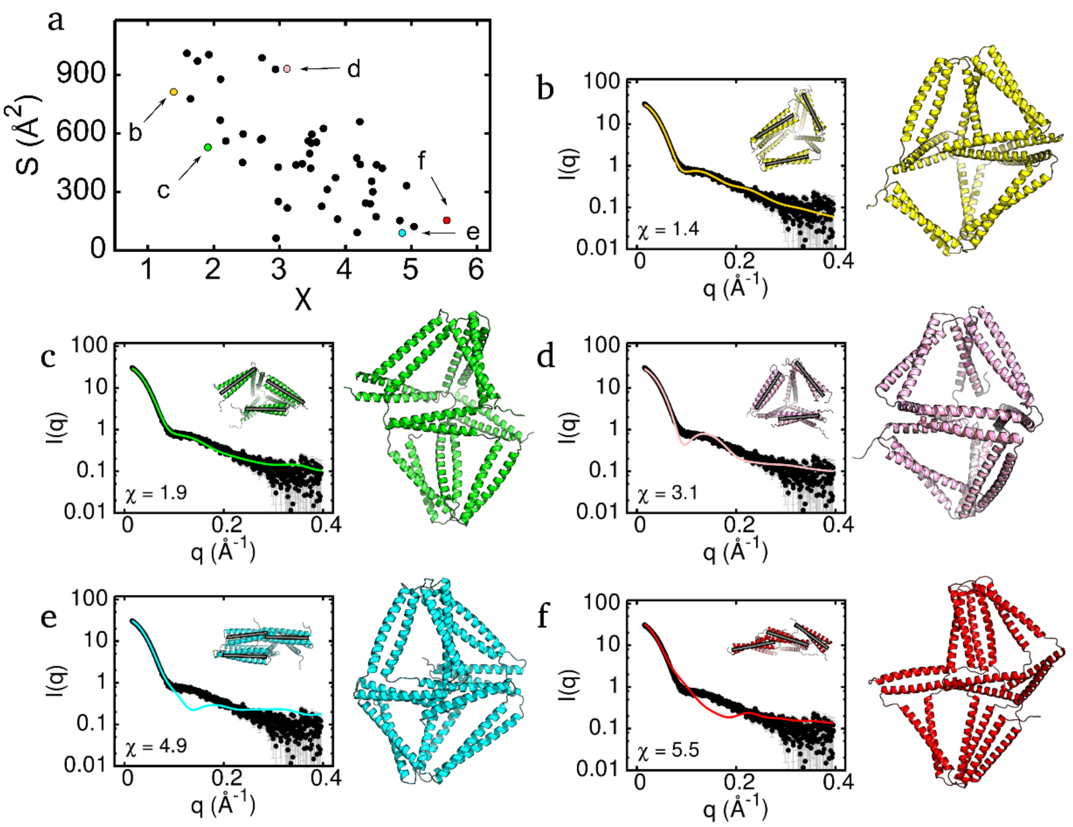

Supplementary Figure 9: SAXS fit to different SBP12$_{9.b}$ models. a, The plot indicates the goodness of SAXS fit (χ) to molecular models in different conformations, which have a different surface area at the interface (*S*). The graph shows a better fit for models having a larger surface area, which corresponds to a larger internal cavity. The data point colour corresponds to the models showed below in the figure. **b-f**, SAXS experimental curve for SBP12$_{9.b}$ (black trace) fitted to the theoretical scattering curve for models in different conformations. Error bars in grey represent the standard deviation for each data point in black (mean). In the plot is shown the fit between experimental data and theoretical scattering curve in addition to the χ value for each conformation. In the top right of each plot is shown the molecular model missing one subunit from bottom view with segments at the interface labelled with bars. The models in different conformations are placed next to the corresponding graphs. Source data are provided as a Source Data file.

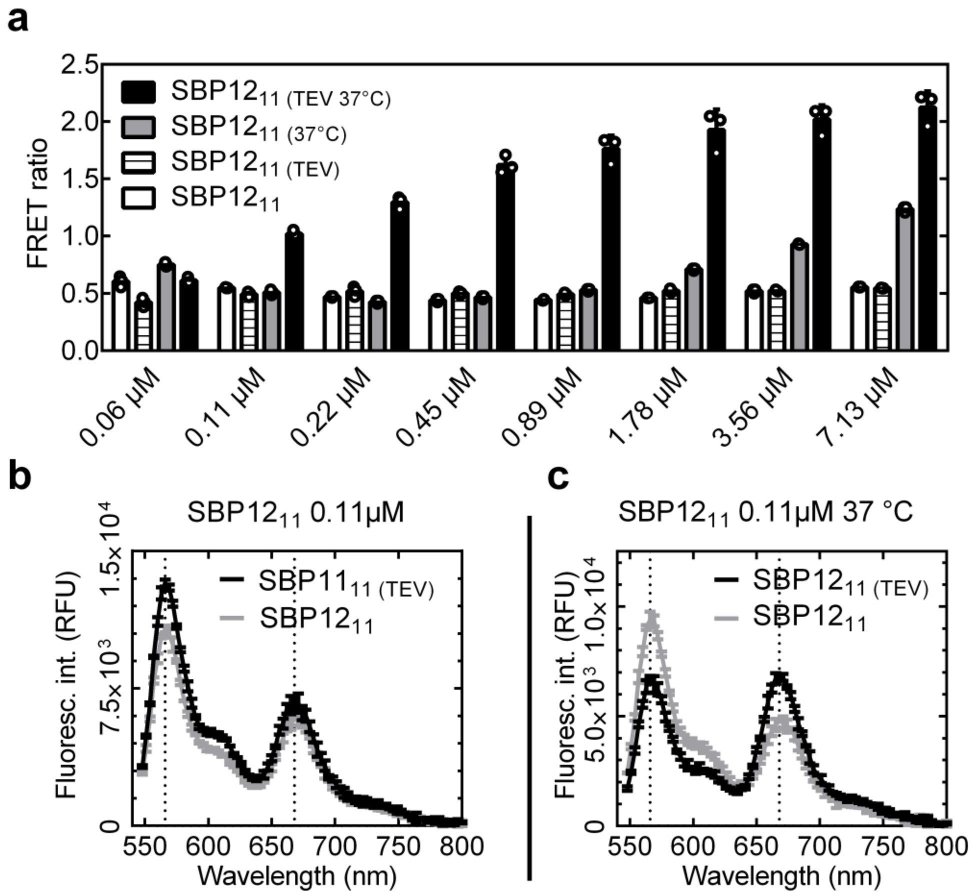

Supplementary Figure 10: FRET ratio and fluorescence spectra of labelled proteins. a, panel shows the FRET ratio at different concentration of the two labelled subunits $SBP1_{11}$ and $SBP2_{11}$ mixed in equimolar ratio (with and without addition of TEV protease and incubation at 37 °C). Error bars represent the standard deviation of three measurements of the same samples (n=3). **b,** Fluorescence spectra of the two subunits $SBP1_{11}$ and $SBP2_{11}$ mixed in equimolar ratio, in the presence or absence of TEV protease. **c**, Fluorescence spectra of the two subunits $SBP1_{11}$ and $SBP2_{11}$ mixed in equimolar ratio, in the presence or absence of TEV protease after incubation at 37 °C. Error bars represent the standard deviation of three measurements of the same samples (n=3). Fluorescence signal is reported in relative fluorescence units (RFU). Source data are provided as a Source Data file.

Supplementary Tables

CC	Orientation	Sequence	Helicity
Register		*gabcdef gabcdef gabcdef gabcdef gab*	
P1SN	Parallel	SPED EIRQLEQ ENSQLER ENQRLEQ EIYQLER	16.18
P2SN	Parallel	SPED KIEELKE KNSQLKE KNEELKQ KIYELKE	6.82
P3SN	Parallel	SPED EIQQLEE EISQLEQ KNSELKE KNQELKY	4.52
P4SN	Parallel	SPED KISQLKE KIQQLKQ ENQQLEE ENSQLEY	3.84
P5SN	Parallel	SPED ENSQLEE KISQLKQ KNSELKE EIQQLEY	3.51
P5SH	Parallel	SPED ENEKLEE KIWELKR KNEELKR EIKELEE	44.47
P6SN	Parallel	SPED KNSELKE EIQQLEE ENQQLEE KISELKY	2.53
P6SH	Parallel	SPED KNEELKR EIKELEW ENEELER KIEELKR	30.73
P7SN	Parallel	SPED EIQQLEE KNSQLKQ EISQLEE KNQELKY	3.54
P7SH	Parallel	SPED EIKELEW KNEELKR EIKELEE KNEELKR	40.02
P8SN	Parallel	SPED KISELKE ENQQLEQ KIQQLKE ENSQLEY	4.28
P8SH	Parallel	SPED KIEELKR ENEELEW KIEELKR ENEELEK	28.09
P9SN	Parallel	SPED ENQSLEQ KNSQLKQ EISQLEQ EIQQLEY	3.06
P9SH	Parallel	SPED ENEKLER KNEELKW EIKKLER EIKELER	31.42
P10SN	Parallel	SPED KNSQLKE ENSQLEE KIEQLKE KIQELKY	4.97
P10SH	Parallel	SPED KNKELKE ENKELEW KIEELKE KIKELKE	17.83
BCR	Antiparallel	DIEQ ELERAKQ SIEELER EVNQERS RMQYLQT RLS	22.16
AP6SH	Antiparallel	SPED KLEEIKR ELEENEW ELEKIER KLEENKR	29.10

Supplementary Table 1: Coiled-coil building-blocks. Orthogonal dimer-forming CC units used in bipyramidal CCPO cages. Polar amino acid residues in *e* and *g* position are coloured in red and blue. The helicity was calculated with AGADIR (ref. 56) for each individual sequence at pH 7, 5 °C and ionic strength 0.1 M.

Data collection parameters

**Radiation source**	Petra III (DESY)		ALS
**Beamline**	EMBL P12		SIBYLS
**Detector**	Pilatus 6M		Pilatus3 2M
**Beam geometry (mm)**	200 µm x 120 µm		165 µm × 130 µm
**Wavelength (nm)**	0.124		0.103
**Sample-detector distance (m)**	3		1.5
**Mode**	**Batch**	**SEC-SAXS**	**SEC-SAXS**
**Column (flow)**		Superdex 200 increase (0.5 ml/min)	Superdex 200 increase (0.5 ml/min)
**Exposure time (s)**	1s (=20 x 0.05 s)	0.995	3
**Temperature (K)**	298	298	298

Overall parameters

	**BIP18SN**	**SBP_{162}**	**SBP_{16}**	**$SBP12_{9.a}$**	**$SBP12_{9.a-SN}$**	**$SBP12_{9.a-56}$**	**$SBP12_{9.a-L}$**	**$SBP12_{11}$**	**$SBP12_{9.b}$**	**$SBP1_{9.a}$**	**$SBP2_{9.a}$**	**$SBP1_{9.b}$**	**$SBP2_{9.b}$**
**R_g from Guinier (nm)**	4.61±0.02	4.07±0.03	4.26±0.01	5.37±0.02	5.41±0.01	4.36±0.02	4.53±0.01	4.13±0.04	4.04±0.03	3.74±0.02	3.69±0.02	3.4±0.02	3.5±0.01
**R_g from PDDF (nm)**	4.6±0.2	4.1±0.1	4.3±0.2	5.3±0.2	5.7±0.1	4.4±0.1	4.6±0.1	4.2±0.1	4.0±0.1	3.7±0.1	3.7±0.1	3.4±0.1	3.5±0.1
**D_{max} (nm)**	12.4±1.0	13.5±1.0	13.0±0.5	20.0±1.0	20.3±1.0	15.5±0.5	15.2±0.5	13.5±1.0	11.8±0.5	13.4±1.0	12.9±0.5	11.3	11.4
**Porod Volume V_P (nm^3)**	435	335	308	328	341	254	285	296	327	139	139	129	138
**DAMMIF excluded volume (nm^3)**	357.7	-	-	423.5	-	-	-	295	305	-	-	-	-
**Molecular weight from V_P (kDa)**	255.9	197.1	181.2	193.0	200.6	149.4	167.6	174.1	192.4	81.8	81.8	74.1	81.2
**Molecular weight from excluded volume (kDa)**	178.9	-	-	211.8	-	-	-	147.5	152.5	-	-	-	-
**Molecular weight from sequence (kDa)**	80.0	79.9	70.6	83.5	81.5	83.7	83.6	81.7	79.7	41.8	41.8	42.2	41.9
**V_r to BIP18SN**	-	3.5	4.4	8.2	8.3	7.6	7.6	4.7	1.8	-	-	-	-

**Software employed**	
**Primary data reduction**	SASFLOW
**Data processing**	PRIMUS
***Ab initio* modelling**	DAMMIF/DAMMIN
**Validation and averaging**	DAMAVER
**Validation fitting to molecular models**	PEPSI-SAXS

**Parameters of filtered *ab initio* model**	**Normalised Spatial Discrepancy**	**Standard deviation of NSD**	**Number of models excluded**	**Final χ^2 after DAMMIN**
**BIP18SN**	0.572	0.026	0	*
**$SBP12_{9.a}$**	0.729	0.031	0	*
**$SBP12_{9.b}$**	1.199	0.035	1	1.52
**$SBP12_{11}$**	1.195	0.035	1	0.95

Supplementary Table 2: SAXS results. The table indicates values resulting from SAXS analysis. The volatility ratio (V_r) was calculated between the SAXS profile of BIP18SN, taken as reference, and the SAXS profile of the sample indicated in the table.* *ab initio* models were generated with DAMMIF

NAME	SEQUENCE
Primers used for cloning constructs starting from the DNA of BIP18SN	
F-SBP_{16}	GATCGTACCATATGAGCCCGGAGGACGAGATTCGCCAACTG
R-SBP_{16}	ATGTCCTTCTCGAGACTTAAGCGGGTTTGCAGGTATTGCATACGGCTACGCTC
F-SBP_{2}	GATCGTACCATATGAGTCCTGAGGATAAGATTAGCCAACTGAACAAG
R-SBP_{2}	ATGTCCTTCTCGAGCTTCAGTTCGCTAATCTTTTCTTCCAGTTGTTG
F-SBP_{15}	CTTTAAGAAGGAGATATACATATGGACATCGAGCAGGAATTAGAGCG
R-SBP_{15}	CATATGTATATCTCCTTCTTAAAGTTAAACAAAATTATTTC
R-$SBP1_{9.b}$	ACCACTTTGGAAATATAAGTTTTCCATATGGTGATGGTGGTGGTGGTGATGCCCCATGGT
Primers used for cloning constructs starting from the DNA of $SBP1_{11}$ and $SBP2_{11}$	
F-$SBP1_{9.b}$	CATCACCATATGGAAAACTTATATTTCCAAAGTGGTTCCGGGTCTCCTGAGGATGAG
F-$SBP2_{9.b}$	CAGAGCGGTAGTGGGTCACTCGAGCACCACCATCACCACCATCACCACTAATGACCTAGG
R-$SBP2_{9.b}$	GATGGTGGTGCTCGAGTGACCCACTACCGCTCTGAAAATACAAATTCTCGCC
F-$pETSBP1_{9.b}$	CTGAAGGAAAAAAATCAGGAGCTTAAGTATCTCGAGTAATGACCTAGGCTGCTAAACAAA
R-$pETSBP1_{9.b}$	GTTTTCTTCCTTCAGTTCCTTGTTCTTGTCCTCAGGAGACCCGGAACCACTTTGGAAATA
F-$SBP2_{9.b}$	GACAAGAACAAGGAACTGAAGGAAGAAAACAAAGAGCTTGAATGG
R-$SBP2_{9.b}$	ATACTTAAGCTCCTGATTTTTTTCCTTCAGCTCACTATTCTTTTG
F-pETstrep	CTGAAGGAAAAAAATCAGGAGCTTAAGTATCTCGAGTAATGACCTAGGCTGCTAAACAAA
R-pETstrep	TCTCGAACTGTGGGTGAGACCAACCCATGGTGTATATCTCCTTCTTAAAGTTAAACAAAA
F-strep	CTGCATGGTCTCACCCACAGTTCGAGAAATCTGCTTCACCCGAGGACAAGAACAAGGAAC
R-strep	TTCTCGAACTGTGGGTGAGACCATGCAGAACCCATATGGGTGTATATCTCCTTCTTAAAG
F-$SBP1_{11Cys}$	GAACGCGAAATCAAAGAATTAGAACGCGGCTGCGGACCAGGGGACATTGAGCAAGAGC
R-$SBP1_{11Cys}$	GCTCTTGCTCAATGTCCCCTGGTCCGCAGCCGCGTTCTAATTCTTTGATTTCGCGTTC
F-$SBP2_{11Cys}$	GCAGTACCTTCAAACCCGCTTGTCAGGTTGTGGCCCAGGCTCTCCTGAAGACAAAATCG
R-$SBP2_{11Cys}$	CGATTTTGTCTTCAGGAGAGCCTGGGCCACAACCTGACAAGCGGGTTTGAAGGTACTGC

Supplementary Table 3: Primers used for cloning. The table shows all the primers used in this study for cloning the constructs that were not purchased as synthetic genes.

List of protein sequences

Name: BIP18SN

Order of segments: P1SN-BCR-P7SN-P2SN- P3SN-P9SN-BCR-P4SN-P5SN-P1SN-BCR-P8SN-P2SN-P3SN-P10SN-BCR-P4SN-P6SN

Amino acid sequence:

MGHHHHHHHHENLYFQGSPEDEIRQLEQENSQLERENQRLEQEIYQLERGSGPGDIEQELERAKQSIEELEREVNQERSRMQYLQTRLSGSGPGSPEDEIQQLEEKNSQLKQEISQLEEKNQELKYGSGPGSPEDKIEELKEKNSQLKEKNEELKQKIYELKEGSGPGSPEDEIQQLEEEISQLEQKNSELKEKNQELKYGSGPGSPEDENQSLEQKNSQLKQEISQLEQEIQQLEYGSGPGDIEQELERAKQSIEELEREVNQERSRMQYLQTRLSGSGPGSPEDKISQLKEKIQQLKQENQQLEEENSQLEYGSGPGSPEDENSQLEEKISQLKQKNSELKEEIQQLEYGSGPGSPEDEIRQLEQENSQLERENQRLEQEIYQLERGSGPGDIEQELERAKQSIEELEREVNQERSRMQYLQTRLSGSGPGSPEDKISELKEENQQLEQKIQQLKEENSQLEYGSGPGSPEDKIEELKEKNSQLKEKNEELKQKIYELKEGSGPGSPEDEIQQLEEEISQLEQKNSELKEKNQELKYGSGPGSPEDKNSQLKEENSQLEEKIEQLKEKIQELKYGSGPGDIEQELERAKQSIEELEREVNQERSRMQYLQTRLSGSGPGSPEDKISQLKEKIQQLKQENQQLEEENSQLEYGSGPGSPEDKNSELKEEIQQLEEENQQLEEKISELKYLE

Name: SBP$_{16}$

Order of segments: P1SN-BCR-P7SN-P2SN- P3SN-P9SN-BCR-P4SN-P5SN-P1SN-BCR-P8SN-P2SN-P3SN-P10SN-BCR

Amino acid sequence:

MSPEDEIRQLEQENSQLERENQRLEQEIYQLERGSGPGDIEQELERAKQSIEELEREVNQERSRMQYLQTRLSGSGPGSPEDEIQQLEEKNSQLKQEISQLEEKNQELKYGSGPGSPEDKIEELKEKNSQLKEKNEELKQKIYELKEGSGPGSPEDEIQQLEEEISQLEQKNSELKEKNQELKYGSGPGSPEDENQSLEQKNSQLKQEISQLEQEIQQLEYGSGPGDIEQELERAKQSIEELEREVNQERSRMQYLQTRLSGSGPGSPEDKISQLKEKIQQLKQENQQLEEENSQLEYGSGPGSPEDENSQLEEKISQLKQKNSELKEEIQQLEYGSGPGSPEDEIRQLEQENSQLERENQRLEQEIYQLERGSGPGDIEQELERAKQSIEELEREVNQERSRMQYLQTRLSGSGPGSPEDKISELKEENQQLEQKIQQLKEENSQLEYGSGPGSPEDKIEELKEKNSQLKEKNEELKQKIYELKEGSGPGSPEDEIQQLEEEISQLEQKNSELKEKNQELKYGSGPGSPEDKNSQLKEENSQLEEKIEQLKEKIQELKYGSGPGDIEQELERAKQSIEELEREVNQERSRMQYLQTRLSLEHHHHHHH

Name: SBP$_{2}$

Order of segments: P4SN-P6SN

Amino acid sequence:

MSPEDKISQLKEKIQQLKQENQQLEEENSQLEYGSGPGSPEDKNSELKEEIQQLEEENQQLEEKISELKLEHHHHHHHH

Name: SBP$_{15}$

Order of segments: BCR-P7SN-P2SN- P3SN-P9SN-BCR-P4SN-P5SN-P1SN-BCR-P8SN-P2SN-P3SN-P10SN-BCR

Amino acid sequence:

MDIEQELERAKQSIEELEREVNQERSRMQYLQTRLSGSGPGSPEDEIQQLEEKNSQLKQEISQLEEKNQELKYGSGPGSPEDKIEELKEKNSQLKEKNEELKQKIYELKEGSGPGSPEDEIQQLEEEISQLEQKNSELKEKNQELKYGSGPGSPEDENQSLEQKNSQLKQEISQLEQEIQQLEYGSGPGDIEQELERAKQSIEEL

EREVNQERSRMQYLQTRLSGSGPGSPEDKISQLKEKIQQLKQENQQLEEENSQLEYGSGPGSPEDENSQLEEKISQLKQKNSELKEEIQQLEYGSGPGSPEDEIRQLEQENSQLERENQRLEQEIYQLERGSGPGDIEQELERAKQSIEELEREVNQERSRMQYLQTRLSGSGPGSPEDKISELKEENQQLEQKIQQLKEENSQLEYGSGPGSPEDKIEELKEKNSQLKEKNEELKQKIYELKEGSGPGSPEDEIQQLEEEISQLEQKNSELKEKNQELKYGSGPGSPEDKNSQLKEENSQLEEKIEQLKEKIQELKYGSGPGDIEQELERAKQSIEELEREVNQERSRMQYLQTRLSLEHHHHHHH

Name: SBP$_3$

Order of segments: P4SN-P6SN-P1SN

Amino acid sequence:

MSPEDKISQLKEKIQQLKQENQQLEEENSQLEYGSGPGSPEDKNSELKEEIQQLEEENQQLEEKISELKYGSGPGSPEDEIRQLEQENSQLERENQRLEQEIYQLERLEHHHHHHH

Name: SBP1$_{9.a}$

Order of segments: P3SN-P9SH-BCR-P4SN-P5SH-P1SN-BCR-P7SH-P2SN

Amino acid sequence:

MHHHHHHHHENLYFQGHMSPEDEIQQLEEEISQLEQKNSELKEKNQELKYGSGPGSPEDENEKLERKNEELKWEIKKLEREIKELERGSGPGDIEQELERAKQSIEELEREVNQERSRMQYLQTRLSGSGPGSPEDKISQLKEKIQQLKQENQQLEEENSQLEYGSGPGSPEDENEKLEEKIWELKRKNEELKREIKELEEGSGPGSPEDEIRQLEQENSQLERENQRLEQEIYQLERGSGPGDIEQELERAKQSIEELEREVNQERSRMQYLQTRLSGSGPGSPEDEIKELEWKNEELKREIKELEEKNEELKRGSGPGSPEDKIEELKEKNSQLKEKNEELKQKIYELKELE

Name: SBP2$_{9.a}$

Order of segments: P3SN-P10SH-BCR-P4SN-P6SH-P1SN-BCR-P8SH-P2SN

Amino acid sequence:

MHHHHHHHHENLYFQGHMSPEDEIQQLEEEISQLEQKNSELKEKNQELKYGSGPGSPEDKNKELKEENKELEWKIEELKEKIKELKEGSGPGDIEQELERAKQSIEELEREVNQERSRMQYLQTRLSGSGPGSPEDKISQLKEKIQQLKQENQQLEEENSQLEYGSGPGSPEDKNEELKREIKELEWENEELERKIEELKRGSGPGSPEDEIRQLEQENSQLERENQRLEQEIYQLERGSGPGDIEQELERAKQSIEELEREVNQERSRMQYLQTRLSGSGPGSPEDKIEELKRENEELEWKIEELKRENEELEKGSGPGSPEDKIEELKEKNSQLKEKNEELKQKIYELKELE

Name: SBP1$_{9.a\text{-}SN}$

Order of segments: P3SN-P9SN-BCR-P4SN-P5SN-P1SN-BCR-P7SN-P2SN

Amino acid sequence:

MHHHHHHHHENLYFQGSPEDEIQQLEEEISQLEQKNSELKEKNQELKYGSGPGSPEDENQSLEQKNSQLKQEISQLEQEIQQLEYGSGPGDIEQELERAKQSIEELEREVNQERSRMQYLQTRLSGSGPGSPEDKISQLKEKIQQLKQENQQLEEENSQLEYGSGPGSPEDENSQLEEKISQLKQKNSELKEEIQQLEYGSGPGSPEDEIRQLEQENSQLERENQRLEQEIYQLERGSGPGDIEQELERAKQSIEELEREVNQERSRMQYLQTRLSGSGPGSPEDEIQQLEEKNSQLKQEISQLEEKNQELKYGSGPGSPEDKIEELKEKNSQLKEKNEELKQKIYELKE

Name: SBP2$_{9.a\text{-}SN}$

Order of segments: P3SN-P10SN-BCR-P4SN-P6SN-P1SN-BCR-P8SN-P2SN

Amino acid sequence:
MHHHHHHHHENLYFQGSPEDEIQQLEEEISQLEQKNSELKEKNQELKYGSGPGSPEDKNSQLKEENS
QLEEKIEQLKEKIQELKYGSGPGDIEQELERAKQSIEELEREVNQERSRMQYLQTRLSGSGPGSPEDKI
SQLKEKIQQLKQENQQLEEENSQLEYGSGPGSPEDKNSELKEEIQQLEEENQQLEEKISELKYGSGPG
SPEDEIRQLEQENSQLERENQRLEQEIYQLERGSGPGDIEQELERAKQSIEELEREVNQERSRMQYLQ
TRLSGSGPGSPEDKISELKEENQQLEQKIQQLKEENSQLEYGSGPGSPEDKIEELKEKNSQLKEKNEEL
KQKIYELKE

Name: SBP1$_{9.a\text{-}56}$
Order of segments: P3SN-P5SH-BCR-P4SN-P5SH-P1SN-BCR-P5SH-P2SN
Amino acid sequence:
MHHHHHHHHENLYFQGHMSPEDEIQQLEEEISQLEQKNSELKEKNQELKYGSGPGSPEDENEKLEE
KIWELKRKNEELKREIKELEEGSGPGDIEQELERAKQSIEELEREVNQERSRMQYLQTRLSGSGPGSP
EDKISQLKEKIQQLKQENQQLEEENSQLEYGSGPGSPEDENEKLEEKIWELKRKNEELKREIKELEEG
SGPGSPEDEIRQLEQENSQLERENQRLEQEIYQLERGSGPGDIEQELERAKQSIEELEREVNQERSRMQ
YLQTRLSGSGPGSPEDENEKLEEKIWELKRKNEELKREIKELEEGSGPGSPEDKIEELKEKNSQLKEK
NEELKQKIYELKELE

Name: SBP2$_{9.a\text{-}56}$
Order of segments: P3SN-AP6SH-BCR-P4SN-P6SH-P1SN-BCR-P6SH-P2SN
Amino acid sequence:
MHHHHHHHHENLYFQGHMSPEDEIQQLEEEISQLEQKNSELKEKNQELKYGSGPGSPEDKLEEIKRE
LEENEWELEKIERKLEENKRGSGPGDIEQELERAKQSIEELEREVNQERSRMQYLQTRLSGSGPGSPE
DKISQLKEKIQQLKQENQQLEEENSQLEYGSGPGSPEDKNEELKREIKELEWENEELERKIEELKRGS
GPGSPEDEIRQLEQENSQLERENQRLEQEIYQLERGSGPGDIEQELERAKQSIEELEREVNQERSRMQ
YLQTRLSGSGPGSPEDKNEELKREIKELEWENEELERKIEELKRGSGPGSPEDKIEELKEKNSQLKEK
NEELKQKIYELKELE

Name: SBP1$_{9.a\text{-}L}$
Order of segments: P3SN-P5SH-BCR-P4SN-P9SH-P1SN-BCR-P7SH-P2SN
Amino acid sequence:
MHHHHHHHHENLYFQGHMSPEDEIQQLEEEISQLEQKNSELKEKNQELKYGSGPGSPEDENEKLEE
KIWELKRKNEELKREIKELEEGSGPGDIEQELERAKQSIEELEREVNQERSRMQYLQTRLSGSGPGSP
EDKISQLKEKIQQLKQENQQLEEENSQLEYGSGPGSPEDENEKLERKNEELKWEIKKLEREIKELERG
SGPGSPEDEIRQLEQENSQLERENQRLEQEIYQLERGSGPGDIEQELERAKQSIEELEREVNQERSRMQ
YLQTRLSGSGPGSPEDEIKELEWKNEELKREIKELEEKNEELKRGSGPGSPEDKIEELKEKNSQLKEK
NEELKQKIYELKELE

Name: SBP2$_{9.a\text{-}L}$
Order of segments P3SN-AP6SH-BCR-P4SN-P8SH-P1SN-BCR-P10SH-P2SN
Amino acid sequence:
MHHHHHHHHENLYFQGHMSPEDEIQQLEEEISQLEQKNSELKEKNQELKYGSGPGSPEDKLEEIKRE
LEENEWELEKIERKLEENKRGSGPGDIEQELERAKQSIEELEREVNQERSRMQYLQTRLSGSGPGSPE
DKISQLKEKIQQLKQENQQLEEENSQLEYGSGPGSPEDKIEELKRENEELEWKIEELKRENEELEKGS
GPGSPEDEIRQLEQENSQLERENQRLEQEIYQLERGSGPGDIEQELERAKQSIEELEREVNQERSRMQ
YLQTRLSGSGPGSPEDKNKELKEENKELEWKIEELKEKIKELKEGSGPGSPEDKIEELKEKNSQLKEK

NEELKQKIYELKELE

Name: $SBP1_{9.b}$
Order of segments: P1SN-P3SN-P9SH-BCR-P4SN-P5SH-P2SN-BCR-P7SH
Amino acid sequence:
MGHHHHHHHHMENLYFQSGSGSPEDEIRQLEQENSQLERENQRLEQEIYQLERGSGPGSPEDEIQQL
EEEISQLEQKNSELKEKNQELKYGSGPGSPEDENEKLERKNEELKWEIKKLEREIKELERGSGPGDIE
QELERAKQSIEELEREVNQERSRMQYLQTRLSGSGPGSPEDKISQLKEKIQQLKQENQQLEEENSQLE
YGSGPGSPEDENEKLEEKIWELKRKNEELKREIKELEEGSGPGSPEDKIEELKEKNSQLKEKNEELKQ
KIYELKEGSGPGDIEQELERAKQSIEELEREVNQERSRMQYLQTRLSGSGPGSPEDEIKELEWKNEEL
KREIKELEEKNEELKRLELE

Name: $SBP2_{9.b}$
Order of segments: P10SH-BCR-P4SN-P6SH-P2SN-BCR-P8SH-P1SN-P3SN
Amino acid sequence:
MGHHHHHHHHMENLYFQSGSGSPEDKNKELKEENKELEWKIEELKEKIKELKEGSGPGDIEQELER
AKQSIEELEREVNQERSRMQYLQTRLSGSGPGSPEDKISQLKEKIQQLKQENQQLEEENSQLEYGSGP
GSPEDKNEELKREIKELEWENEELERKIEELKRGSGPGSPEDKIEELKEKNSQLKEKNEELKQKIYEL
KEGSGPGDIEQELERAKQSIEELEREVNQERSRMQYLQTRLSGSGPGSPEDKIEELKRENEELEWKIE
ELKRENEELEKGSGPGSPEDEIRQLEQENSQLERENQRLEQEIYQLERGSGPGSPEDEIQQLEEEISQLE
QKNSELKEKNQELKYLE

Name: $SBP1_{11}$
Order of segments: P10SH-P8SH-TEVs-P1SN-P3SN-P9SH-**Cys**-BCR-P4SN-P5SH-P2SN-BCR-P7SH
Amino acid sequence:
MGHHHHHHHHMSPEDKNKELKEENKELEWKIEELKEKIKELKEGSGPGSPEDKIEELKRENEELEW
KIEELKRENEELEKGSGENLYFQSGSGSPEDEIRQLEQENSQLERENQRLEQEIYQLERGSGPGSPEDE
IQQLEEEISQLEQKNSELKEKNQELKYGSGPGSPEDENEKLERKNEELKWEIKKLEREIKELERG**C**GP
GDIEQELERAKQSIEELEREVNQERSRMQYLQTRLSGSGPGSPEDKISQLKEKIQQLKQENQQLEEEN
SQLEYGSGPGSPEDENEKLEEKIWELKRKNEELKREIKELEEGSGPGSPEDKIEELKEKNSQLKEKNE
ELKQKIYELKEGSGPGDIEQELERAKQSIEELEREVNQERSRMQYLQTRLSGSGPGSPEDEIKELEWK
NEELKREIKELEEKNEELKRLELE

Name: $SBP2_{11}$
Order of segments: P10SH-BCR-P4SN-P6SH-P2SN-BCR-**Cys**-P8SH-P1SN-P3SN-TEVs-P9SH-P7SH
Amino acid sequence:
MGSAWSHPQFEKSASPEDKNKELKEENKELEWKIEELKEKIKELKEGSGPGDIEQELERAKQSIEELE
REVNQERSRMQYLQTRLSGSGPGSPEDKISQLKEKIQQLKQENQQLEEENSQLEYGSGPGSPEDKNE
ELKREIKELEWENEELERKIEELKRGSGPGSPEDKIEELKEKNSQLKEKNEELKQKIYELKEGSGPGDI
EQELERAKQSIEELEREVNQERSRMQYLQTRLSG**C**GPGSPEDKIEELKRENEELEWKIEELKRENEEL
EKGSGPGSPEDEIRQLEQENSQLERENQRLEQEIYQLERGSGPGSPEDEIQQLEEEISQLEQKNSELKE
KNQELKYGSGENLYFQSGSGSPEDENEKLERKNEELKWEIKKLEREIKELERGSGPGSPEDEIKELEW
KNEELKREIKELEEKNEELKRLEHHHHHHHH

Name: TEV protease

Amino acid sequence:

MGHHHHHHHHGESLFKGPRDYNPISSTICHLTNESDGHTTSLYGIGFGPFIITNKHLFRRNNGTLLVQ
SLHGVFKVKNTTTLQQHLIDGRDMIIIRMPKDFPPFPQKLKFREPQREERICLVTTNFQTKSMSSMVS
DTSCTFPSSDGIFWKHWIQTKDGQCGSPLVSTRDGFIVGIHSASNFTNTNNYFTSVPKNFMELLTNQE
AQQWVSGWRLNADSVLWGGHKVFMSKPEEPFQPVKEATQLMSELVYSQ

Affinity tags are underlined

List of DNA sequences

Name: BIP18SN

DNA sequence:

CATATGGGCCACCACCATCATCACCATCACCACGAAAATCTGTACTTTCAAGGCAGCCCGGAGGACGAGATTCGCCAACTG
GAGCAGGAGAACAGTCAACTGGAACGTGAAAACCAGCGTCTGGAACAAGAAATTTATCAGCTGGAACGCGGTAGCGGCC
CTGGTGACATCGAGCAGGAATTAGAGCGCGCAAAACAGAGTATCGAAGAGCTGGAGCGCGAGGTGAACCAAGAACGCAG
TCGTATGCAATACCTGCAGACACGCTTAAGCGGTAGCGGTCCGGGTAGCCCGGAAGACGAAATTCAACAACTGGAGGAAA
AGAATAGTCAGCTGAAGCAGGAGATTAGTCAACTGGAAGAGAAAAATCAAGAGCTGAAATATGGCAGTGGTCCGGGCAGC
CCGGAAGATAAAATCGAGGAGCTGAAGGAGAAAAATAGCCAGCTGAAAGAAAAGAATGAAGAACTGAAACAGAAGATTT
ACGAGCTGAAGGAAGGCAGTGGCCCGGGCAGCCCGGAGGACGAGATCCAGCAGCTGGAAGAGGAGATTAGCCAGCTGG
AGCAGAAGAATAGCGAGCTGAAGGAGAAGAACCAAGAGTTAAAGTATGGCAGCGGTCCGGGTAGCCCGGAAGATGAAAA
TCAGAGCCTGGAGCAAAAGAACAGCCAACTGAAGCAGGAAATTAGCCAATTAGAGCAGGAGATCCAACAACTGGAGTAT
GGCAGTGGCCCGGGCGACATTGAGCAAGAACTGGAACGCGCCAAGCAGAGCATTGAAGAGCTGGAACGCGAAGTGAATC
AGGAGCGCAGCCGCATGCAGTATCTGCAGACCCGTCTGAGCGGTAGTGGCCCGGGCAGCCCGGAGGACAAGATCAGCCA
ACTGAAGGAGAAGATCCAGCAGCTGAAACAAGAAAACCAGCAACTGGAGGAAGAAAATAGCCAACTGGAATATGGTAGC
GGTCCGGGTAGCCCGGAAGATGAAAATAGTCAGTTAGAGGAAAAAATTAGCCAATTAAAACAGAAAAATAGCGAACTGAA
GGAAGAAATTCAACAGCTGGAATACGGCAGTGGTCCTGGTAGTCCGGAGGACGAAATTCGTCAGCTGGAACAGGAAAAC
AGCCAGCTGGAACGTGAGAACCAGCGCCTGGAGCAAGAGATTTACCAGCTGGAACGTGGCAGCGGTCCTGGTGACATTG
AACAGGAACTGGAGCGCGCCAAACAGAGCATCGAGGAGCTGGAGCGCGAGGTGAACCAGGAGCGTAGCCGCATGCAATA
TCTGCAGACCCGCCTGAGTGGTAGTGGTCCGGGCAGCCCTGAAGACAAAATCAGCGAACTGAAAGAAGAAAACCAGCAA
CTGGAGCAAAAAATCCAGCAGCTGAAAGAAGAAAATAGTCAACTGGAGTACGGTAGCGGTCCGGGCAGTCCGGAAGACA
AGATCGAGGAGCTGAAAGAGAAAAATAGCCAACTGAAAGAGAAAAACGAAGAACTGAAGCAGAAAATTTATGAGCTGAA
AGAAGGTAGCGGCCCTGGTAGTCCGGAGGATGAGATTCAGCAACTGGAAGAGGAGATTAGCCAATTAGAACAGAAAAATA
GTGAACTGAAGGAAAAGAATCAGGAATTAAAATACGGCAGCGGTCCGGGCAGTCCTGAAGATAAAAACAGTCAACTGAA
GGAAGAGAACAGCCAGCTGGAAGAGAAGATCGAGCAACTGAAAGAGAAGATCCAGGAGCTGAAGTATGGTAGCGGCCCG
GGCGATATTGAACAGGAACTGGAACGCGCAAAGCAGAGCATCGAAGAGCTGGAACGCGAAGTGAACCAAGAGCGTAGCC
GTATGCAATACCTGCAAACCCGCTTAAGTGGTAGCGGTCCGGGCAGTCCTGAGGATAAGATTAGCCAACTGAAAGAAAAG
ATTCAGCAACTGAAACAAGAGAATCAACAGCTGGAGGAAGAGAATAGCCAGCTGGAGTACGGTAGCGGCCCGGGTAGCC
CGGAGGATAAGAACAGTGAGCTGAAGGAAGAAATCCAGCAACTGGAAGAAGAAAATCAACAACTGGAAGAAAAGATTAG
CGAACTGAAGTATTAATGACTCGAG

Name: SBP16

DNA sequence:

GATCGTACCATATGAGCCCGGAGGACGAGATTCGCCAACTGGAGCAGGAGAACAGTCAACTGGAACGTGAAAACCAGCGT
CTGGAACAAGAAATTTATCAGCTGGAACGCGGTAGCGGCCCTGGTGACATCGAGCAGGAATTAGAGCGCGCAAAACAGAG
TATCGAAGAGCTGGAGCGCGAGGTGAACCAAGAACGCAGTCGTATGCAATACCTGCAGACACGCTTAAGCGGTAGCGGTC
CGGGTAGCCCGGAAGACGAAATTCAACAACTGGAGGAAAAGAATAGTCAGCTGAAGCAGGAGATTAGTCAACTGGAAGA
GAAAAATCAAGAGCTGAAATATGGCAGTGGTCCGGGCAGCCCGGAAGATAAAATCGAGGAGCTGAAGGAGAAAAATAGC
CAGCTGAAAGAAAAGAATGAAGAACTGAAACAGAAGATTTACGAGCTGAAGGAAGGCAGTGGCCCGGGCAGCCCGGAG
GACGAGATCCAGCAGCTGGAAGAGGAGATTAGCCAGCTGGAGCAGAAGAATAGCGAGCTGAAGGAGAAGAACCAAGAG
TTAAAGTATGGCAGCGGTCCGGGTAGCCCGGAAGATGAAAATCAGAGCCTGGAGCAAAAGAACAGCCAACTGAAGCAGG
AAATTAGCCAATTAGAGCAGGAGATCCAACAACTGGAGTATGGCAGTGGCCCGGGCGACATTGAGCAAGAACTGGAACGC
GCCAAGCAGAGCATTGAAGAGCTGGAACGCGAAGTGAATCAGGAGCGCAGCCGCATGCAGTATCTGCAGACCCGTCTGAG
CGGTAGTGGCCCGGGCAGCCCGGAGGACAAGATCAGCCAACTGAAGGAGAAGATCCAGCAGCTGAAACAAGAAAACCA
GCAACTGGAGGAAGAAAATAGCCAACTGGAATATGGTAGCGGTCCGGGTAGCCCGGAAGATGAAAATAGTCAGTTAGAGG
AAAAAATTAGCCAATTAAAACAGAAAAATAGCGAACTGAAGGAAGAAATTCAACAGCTGGAATACGGCAGTGGTCCTGGT
AGTCCGGAGGACGAAATTCGTCAGCTGGAACAGGAAAACAGCCAGCTGGAACGTGAGAACCAGCGCCTGGAGCAAGAG
ATTTACCAGCTGGAACGTGGCAGCGGTCCTGGTGACATTGAACAGGAACTGGAGCGCGCCAAACAGAGCATCGAGGAGCT
GGAGCGCGAGGTGAACCAGGAGCGTAGCCGCATGCAATATCTGCAGACCCGCCTGAGTGGTAGTGGTCCGGGCAGCCCTG
AAGACAAAATCAGCGAACTGAAAGAAGAAAACCAGCAACTGGAGCAAAAAATCCAGCAGCTGAAAGAAGAAAATAGTC
AACTGGAGTACGGTAGCGGTCCGGGCAGTCCGGAAGACAAGATCGAGGAGCTGAAAGAGAAAAATAGCCAACTGAAAGA
GAAAAACGAAGAACTGAAGCAGAAAATTTATGAGCTGAAAGAAGGTAGCGGCCCTGGTAGTCCGGAGGATGAGATTCAG
CAACTGGAAGAGGAGATTAGCCAATTAGAACAGAAAAATAGTGAACTGAAGGAAAAGAATCAGGAATTAAAATACGGCAG
CGGTCCGGGCAGTCCTGAAGATAAAAACAGTCAACTGAAGGAAGAGAACAGCCAGCTGGAAGAGAAGATCGAGCAACTG
AAAGAGAAGATCCAGGAGCTGAAGTATGGTAGCGGCCCGGGCGATATTGAACAGGAACTGGAACGCGCAAAGCAGAGCA

TCGAAGAGCTGGAACGCGAAGTGAACCAAGAGCGTAGCCGTATGCAATACCTGCAAACCCGCTTAAGTCTCGAGAAGGAC
AT

Name: SBP_2

DNA sequence:

GATCGTACCATATGAGTCCTGAGGATAAGATTAGCCAACTGAAAGAAAAGATTCAGCAACTGAAACAAGAGAATCAACAG
CTGGAGGAAGAGAATAGCCAGCTGGAGTACGGTAGCGGCCCGGGTAGCCCGGAGGATAAGAACAGTGAGCTGAAGGAAG
AAATCCAGCAACTGGAAGAAGAAAATCAACAACTGGAAGAAAAGATTAGCGAACTGAAGCTCGAGAAGGACAT

Name: SBP_{15}

DNA sequence:

GAAGGAGATATACATATGGACATCGAGCAGGAATTAGAGCGCGCAAAACAGAGTATCGAAGAGCTGGAGCGCGAGGTGAA
CCAAGAACGCAGTCGTATGCAATACCTGCAGACACGCTTAAGCGGTAGCGGTCCGGGTAGCCCGGAAGACGAAATTCAAC
AACTGGAGGAAAAGAATAGTCAGCTGAAGCAGGAGATTAGTCAACTGGAAGAGAAAAATCAAGAGCTGAAATATGGCAG
TGGTCCGGGCAGCCCGGAAGATAAAATCGAGGAGCTGAAGGAGAAAAATAGCCAGCTGAAAGAAAAGAATGAAGAACTG
AAACAGAAGATTTACGAGCTGAAGGAAGGCAGTGGCCCGGGCAGCCCGGAGGACGAGATCCAGCAGCTGGAAGAGGAG
ATTAGCCAGCTGGAGCAGAAGAATAGCGAGCTGAAGGAGAAGAACCAAGAGTTAAAGTATGGCAGCGGTCCGGGTAGCC
CGGAAGATGAAAATCAGAGCCTGGAGCAAAAGAACAGCCAACTGAAGCAGGAAATTAGCCAATTAGAGCAGGAGATCCA
ACAACTGGAGTATGGCAGTGGCCCGGGCGACATTGAGCAAGAACTGGAACGCGCCAAGCAGAGCATTGAAGAGCTGGAA
CGCGAAGTGAATCAGGAGCGCAGCCGCATGCAGTATCTGCAGACCCGTCTGAGCGGTAGTGGCCCGGGCAGCCCGGAGGA
CAAGATCAGCCAACTGAAGGAGAAGATCCAGCAGCTGAAACAAGAAAACCAGCAACTGGAGGAAGAAAATAGCCAACTG
GAATATGGTAGCGGTCCGGGTAGCCCGGAAGATGAAAATAGTCAGTTAGAGGAAAAAATTAGCCAATTAAAACAGAAAAA
TAGCGAACTGAAGGAAGAAATTCAACAGCTGGAATACGGCAGTGGTCCTGGTAGTCCGGAGGACGAAATTCGTCAGCTGG
AACAGGAAAACAGCCAGCTGGAACGTGAGAACCAGCGCCTGGAGCAAGAGATTTACCAGCTGGAACGTGGCAGCGGTCC
TGGTGACATTGAACAGGAACTGGAGCGCGCCAAACAGAGCATCGAGGAGCTGGAGCGCGAGGTGAACCAGGAGCGTAGC
CGCATGCAATATCTGCAGACCCGCCTGAGTGGTAGTGGTCCGGGCAGCCCTGAAGACAAAATCAGCGAACTGAAAGAAGA
AAACCAGCAACTGGAGCAAAAAATCCAGCAGCTGAAAGAAGAAAATAGTCAACTGGAGTACGGTAGCGGTCCGGGCAGT
CCGGAAGACAAGATCGAGGAGCTGAAAGAGAAAAATAGCCAACTGAAAGAGAAAAACGAAGAACTGAAGCAGAAAATT
TATGAGCTGAAAGAAGGTAGCGGCCCTGGTAGTCCGGAGGATGAGATTCAGCAACTGGAAGAGGAGATTAGCCAATTAGA
ACAGAAAAATAGTGAACTGAAGGAAAAGAATCAGGAATTAAAATACGGCAGCGGTCCGGGCAGTCCTGAAGATAAAAAC
AGTCAACTGAAGGAAGAGAACAGCCAGCTGGAAGAGAAGATCGAGCAACTGAAAGAGAAGATCCAGGAGCTGAAGTAT
GGTAGCGGCCCGGGCGATATTGAACAGGAACTGGAACGCGCAAAGCAGAGCATCGAAGAGCTGGAACGCGAAGTGAACC
AAGAGCGTAGCCGTATGCAATACCTGCAAACCCGCTTAAGTCTCGAGCACCACCACCACCACCACCACCACTAATTGATTA
ATACCTAGG

Name: SBP_3

DNA sequence:

TCTAGAAATAATTTTGTTTAACTTTAAGAAGGAGATATACATATGTCCCCAGAGGATAAAATCAGTCAGTTAAAGGAGAAGA
TCCAGCAATTAAAACAAGAAAACCAACAGCTTGAAGAGGAAAACAGCCAGCTGGAATATGGTTCTGGTCCCGGCTCCCCC
GAAGACAAAAACTCGGAATTAAAGGAAGAAATCCAGCAGCTGGAAGAGGAGAATCAGCAACTGGAAGAAAAAATTTCCG
AATTGAAGTATGGAAGCGGACCAGGCTCACCTGAAGACGAAATTCGCCAGCTTGAACAAGAGAACAGCCAATTAGAACGC
GAAAATCAACGTCTGGAGCAGGAGATCTACCAGTTGGAGCGTCTCGAGCACCACCACCACCACCACCACCACTAATTGATT
AATACCTAGG

Name: $SBP1_{9.a}$

DNA sequence:

TCTAGAAATAATTTTGTTTAACTTTAAGAAGGAGATATACACATGCACCATCACCATCATCACCATCACGAGAATCTTTACTT
CCAAGGCCATATGAGTCCCGAAGATGAAATTCAACAACTGGAGGAAGAGATTTCTCAACTTGAGCAGAAAAACTCAGAAT
TAAAAGAAAAAAACCAAGAGCTGAAGTACGGTAGTGGCCCGGGTTCACCTGAAGACGAGAATGAAAAATTGGAGCGCAA
GAACGAGGAATTAAAGTGGGAAATCAAGAAGCTGGAACGTGAAATCAAGGAATTGGAGCGTGGGAGTGGACCGGGGGAC
ATCGAACAAGAATTGGAACGCGCGAAGCAGTCGATTGAGGAACTTGAACGCGAAGTTAATCAGGAGCGTTCACGTATGCA
GTATCTTCAAACGCGCTTGTCGGGGTCGGGGCCAGGTAGTCCTGAAGACAAGATCTCCCAGTTAAAGGAAAAAATTCAAC
AATTGAAGCAGGAAAATCAGCAGTTGGAAGAGGAAAATTCTCAGTTAGAGTACGGGAGCGGACCAGGGTCCCCTGAGGA
CGAAAACGAGAAATTAGAAGAGAAGATCTGGGAGTTAAAACGTAAGAATGAAGAGTTAAAGCGCGAGATCAAGGAGCTG
GAAGAAGGTTCCGGCCCCGGAAGTCCTGAGGACGAGATTCGCCAGCTGGAGCAGGAGAACTCTCAGCTGGAGCGTGAGA

ACCAGCGTTTAGAACAAGAGATTTACCAGTTAGAGCGTGGCTCTGGTCCTGGGGACATTGAACAGGAACTGGAGCGCGCC
AAGCAATCCATTGAAGAACTGGAGCGTGAGGTGAATCAAGAACGCAGCCGTATGCAGTATTTGCAGACGCGTTTAAGCGG
AAGTGGACCAGGATCACCCGAGGACGAGATTAAAGAGTTGGAGTGGAAAAATGAAGAACTTAAGCGCGAAATTAAAGAG
CTGGAGGAAAAAAACGAGGAATTGAAACGCGGGTCAGGACCCGGTTCACCAGAAGACAAGATTGAAGAACTGAAGGAA
AAAAACAGTCAATTAAAAGAAAAGAATGAGGAGCTGAAGCAGAAGATCTACGAACTGAAAGAGCTCGAGTAATGACCTA
GG

Name: SBP2$_{9.a}$

DNA sequence:

TCTAGAAATAATTTTGTTTAACTTTAAGAAGGAGATATACACATGCATCACCACCACCACCATCACCATGAAAACCTATATTT
TCAGGGACATATGAGTCCTGAGGACGAAATACAGCAGCTTGAGGAAGAAATTTCACAACTTGAGCAGAAGAATTCTGAAT
TGAAGGAAAAGAATCAAGAACTTAAATATGGTTCAGGACCTGGCTCGCCCGAGGATAAGAACAAAGAGTTAAAAGAGGA
AAACAAAGAGCTAGAGTGGAAAATAGAGGAGTTGAAGGAGAAAATCAAAGAATTGAAGGAAGGATCTGGCCCTGGAGAC
ATAGAGCAAGAGTTGGAGAGAGCCAAACAATCGATCGAAGAATTAGAGAGAGAGGTAAATCAAGAGAGATCACGTATGCA
ATACCTGCAAACCAGACTTTCTGGGAGTGGACCTGGATCTCCCGAGGACAAGATATCTCAGTTAAAAGAGAAAATCCAGCA
ATTAAAACAAGAGAACCAGCAGCTGGAAGAAGAGAATTCGCAGCTTGAGTACGGCTCAGGACCAGGTTCCCCCGAGGATA
AGAATGAAGAGCTGAAACGTGAGATTAAAGAGCTGGAGTGGGAAAACGAGGAACTAGAGAGAAAGATAGAAGAGCTGA
AACGAGGGTCTGGTCCCGGAAGTCCTGAGGACGAGATACGACAGTTGGAACAGGAAAACTCTCAACTAGAACGTGAGAA
CCAGAGGTTGGAACAGGAAATCTATCAATTGGAGCGTGGATCTGGACCTGGGGACATCGAGCAGGAACTTGAGAGAGCAA
AGCAATCCATAGAGGAATTAGAAAGGGAAGTTAACCAAGAGAGGTCGCGAATGCAATATTTGCAGACCCGACTTTCTGGCT
CAGGCCCTGGCTCACCAGAGGATAAGATCGAGGAACTAAAACGAGAGAACGAGGAGCTAGAGTGGAAGATTGAGGAACT
GAAGAGGGAAAATGAGGAGCTTGAGAAGGGAAGCGGTCCAGGCTCTCCAGAGGACAAAATAGAAGAGCTCAAGGAAAA
GAATAGCCAATTGAAGGAGAAGAACGAAGAGTTAAAGCAGAAAATCTACGAGTTGAAGGAACTCGAGTAATGACCTAGG

Name: SBP1$_{9.a\text{-}SN}$

DNA sequence:

CATATGCATCACCACCATCACCATCATCACGAGAACCTGTACTTCCAGGGTAGTCCTGAAGATGAGATTCAGCAGCTGGAAG
AAGAAATCAGTCAGCTGGAACAAAAGAATAGCGAGCTGAAAGAGAAAAATCAGGAACTGAAATACGGTAGTGGCCCGGG
TAGCCCGGAGGACGAGAATCAGAGCCTGGAGCAAAAGAATAGTCAACTGAAACAAGAGATTAGCCAACTGGAGCAGGAA
ATTCAACAGCTGGAATACGGCAGCGGCCCGGGTGACATCGAGCAAGAGCTGGAGCGCGCAAAACAGAGCATTGAAGAGC
TGGAACGCGAGGTGAACCAAGAACGTAGCCGTATGCAGTACCTGCAGACCCGTCTGAGCGGCAGCGGCCCGGGTAGCCCG
GAGGACAAAATCAGCCAGCTGAAGGAGAAGATTCAGCAACTGAAGCAAGAAAATCAACAGCTGGAAGAAGAAAACAGC
CAGCTGGAGTATGGTAGTGGCCCGGGTAGTCCGGAGGATGAGAATAGCCAACTGGAGGAAAAGATCAGCCAACTGAAGCA
GAAGAACAGCGAACTGAAGGAAGAGATTCAGCAACTGGAATATGGCAGCGGTCCGGGTAGCCCGGAGGACGAAATTCGC
CAACTGGAGCAAGAGAACAGCCAACTGGAGCGCGAGAATCAGCGTCTGGAACAGGAGATTTACCAACTGGAGCGCGGTA
GCGGTCCGGGTGACATTGAACAGGAACTGGAGCGCGCAAAGCAGAGCATCGAAGAACTGGAGCGTGAAGTGAATCAGGA
ACGCAGCCGCATGCAATATCTGCAGACACGTTTAAGTGGCAGCGGTCCTGGTAGTCCTGAGGACGAGATCCAACAGCTGG
AGGAAAAGAATAGCCAGCTGAAGCAGGAAATTAGCCAGCTGGAGGAGAAGAACCAGGAGCTGAAGTACGGCAGCGGTCC
GGGTAGCCCGGAAGATAAAATCGAGGAGCTGAAAGAGAAAAATAGTCAACTGAAGGAGAAAAATGAAGAACTGAAGCAG
AAAATTTATGAATTAAAAGAATAATGACTCGAG

Name: SBP2$_{9.a\text{-}SN}$

DNA sequence:

GATATACCATGGGCCATCACCATCATCATCACCATCATGAGAACCTGTATTTTCAAGGCCATATGAGTCCTGAAGATGAGATT
CAGCAGCTGGAGGAAGAAATTAGCCAACTGGAACAGAAAAACAGCGAACTGAAGGAGAAAAATCAGGAACTGAAATATG
GCAGCGGCCCGGGTAGCCCGGAGGACAAAAACAGTCAACTGAAAGAAGAGAACAGCCAGCTGGAAGAGAAAATTGAAC
AGTTAAAGGAAAAAATCCAGGAGTTAAAATATGGTAGCGGCCCGGGCGATATTGAACAAGAGCTGGAACGCGCCAAGCAG
AGCATCGAAGAGCTGGAACGCGAAGTGAATCAGGAACGTAGCCGCATGCAATACCTGCAGACCCGCCTGAGTGGCAGCGG
TCCGGGTAGTCCGGAGGACAAAATCAGCCAGCTGAAGGAAAAAATTCAGCAGCTGAAACAGGAAAATCAACAACTGGAA
GAAGAAAATAGCCAGCTGGAATATGGCAGTGGTCCGGGCAGTCCGGAAGATAAAAATAGCGAGCTGAAAGAAGAAATCCA
GCAACTGGAGGAAGAGAATCAGCAGCTGGAGGAAAAGATCAGCGAGCTGAAGTATGGCAGCGGCCCGGGTAGCCCGGAG
GACGAAATTCGCCAGCTGGAGCAGGAAAATAGCCAGCTGGAGCGCGAAAATCAGCGCCTGGAACAAGAAATCTACCAGC
TGGAGCGCGGTAGTGGCCCGGGTGATATTGAGCAGGAACTGGAGCGTGCCAAACAAAGCATTGAGGAACTGGAGCGCGA
GGTGAACCAGGAACGCAGCCGCATGCAATATCTGCAGACACGTTTAAGTGGTAGCGGTCCGGGTAGTCCGGAGGATAAGA
TCAGTGAGTTAAAAGAAGAAAACCAACAGCTGGAACAGAAGATTCAACAACTGAAAGAAGAAAATAGCCAACTGGAATA
CGGCAGTGGCCCGGGCAGCCCGGAAGACAAAATCGAGGAACTGAAAGAGAAGAACAGCCAGCTGAAAGAGAAAAATGA
AGAACTGAAGCAGAAAATCTATGAACTGAAGGAGTAATAGCTCGAGTACCTAGGCTG

Name: SBP1$_{9.a\text{-}56}$

DNA sequence:

TCTAGAAACAACTTTGTGTAATTATGAGAGGGGGACATTCACATGCATCATCATCATCACCATCACCACGAGAACTTATATTT
TCAGGGGCATATGTCTCCAGAAGACGAGATCCAGCAACTTGAAGAAGAGATCAGTCAGTTAGAGCAGAAAAATTCCGAAC
TGAAGGAGAAAAATCAAGAGTTAAAGTACGGCTCCGGTCCAGGTTCACCTGAGGATGAAAACGAGAAATTAGAAGAGAA
GATCTGGGAGTTAAAACGCAAAAATGAAGAGTTGAAGCGTGAGATCAAGGAACTTGAGGAAGGTTCCGGTCCGGGAGAT
ATCGAACAGGAATTAGAGCGCGCAAAGCAATCTATCGAAGAGCTTGAACGTGAGGTAAATCAAGAACGCTCCCGCATGCA
GTACTTGCAAACGCGTTTGTCTGGAAGCGGCCCGGGTAGTCCTGAGGATAAGATTAGTCAATTAAAAGAAAAAATCCAGCA
ATTAAAGCAAGAGAACCAACAATTGGAAGAAGAAAATTCCCAGCTTGAGTACGGGTCAGGCCCTGGGAGCCCAGAGGAC
GAGAATGAAAAATTGGAGGAAAAGATTTGGGAATTGAAACGCAAAAATGAGGAGTTGAAACGTGAGATCAAGGAATTAG
AAGAAGGTAGTGGACCGGGGAGTCCTGAGGACGAAATTCGCCAGCTGGAGCAAGAAAACAGTCAACTGGAGCGCGAAA
ATCAGCGTCTGGAACAGGAGATTTACCAGTTGGAGCGCGGCTCCGGACCGGGTGATATCGAGCAGGAGTTGGAGCGTGCG
AAACAATCGATCGAGGAATTAGAACGCGAGGTGAACCAGGAACGCTCTCGCATGCAGTATTTACAGACCCGTTTGTCAGGG
AGTGGACCAGGCTCACCAGAAGACGAGAACGAAAAGTTGGAAGAGAAAATCTGGGAACTTAAACGTAAGAATGAAGAAT
TAAAGCGTGAGATTAAAGAATTAGAGGAGGGATCAGGACCAGGCAGTCCTGAAGACAAGATCGAAGAACTTAAAGAGAA
GAACAGTCAGCTGAAGGAAAAAAACGAAGAGCTGAAACAGAAGATTTATGAACTGAAAGAACTCGAGTAATGACCTAGG

Name: SBP2$_{9.a\text{-}56}$

DNA sequence:

TCTAGAAACAACTTTGTCTAACTGTAAGAGGGTGATATTCACATGCATCATCATCATCATCACCATCATGAAAATTTGTACTT
CCAAGGACATATGTCTCCCGAGGATGAGATCCAGCAGTTGGAAGAGGAGATCTCACAGTTGGAACAAAAGAACTCTGAGT
TAAAAGAAAAAAACCAAGAATTGAAATATGGCTCTGGCCCGGGTTCTCCAGAAGACAAATTGGAAGAAATTAAACGCGAG
TTGGAAGAGAATGAATGGGAGTTGGAAAAAATCGAGCGTAAGCTTGAAGAAAATAAGCGCGGGAGCGGTCCCGGCGACA
TTGAACAGGAGCTGGAACGTGCAAAGCAATCTATCGAGGAATTAGAGCGCGAAGTGAATCAAGAGCGTAGCCGCATGCAG
TACCTGCAGACACGTTTAAGTGGGTCTGGTCCTGGTTCGCCCGAAGACAAAATTTCGCAGTTAAAGGAAAAGATCCAACA
ACTGAAGCAGGAGAATCAACAGCTGGAGGAAGAGAACAGTCAGTTGGAGTATGGGAGCGGGCCAGGAAGTCCAGAGGA
CAAAAATGAGGAGTTGAAGCGTGAGATCAAAGAATTGGAGTGGGAAAATGAGGAGTTAGAACGTAAGATCGAAGAACTG
AAGCGCGGGTCAGGTCCAGGCTCCCCTGAAGATGAGATCCGTCAGCTTGAACAAGAAAATTCGCAGTTGGAGCGCGAGA
ACCAACGCTTAGAGCAAGAGATTTACCAACTTGAACGTGGATCAGGACCTGGGGACATCGAGCAAGAGCTTGAACGCGCG
AAACAGTCAATTGAGGAATTAGAACGCGAGGTAAACCAAGAACGCTCCCGTATGCAGTACTTGCAGACGCGCCTTTCGGG
TAGCGGACCCGGTTCCCCAGAAGATAAAAATGAAGAGCTTAAACGCGAAATTAAAGAACTTGAGTGGGAGAACGAGGAAT
TAGAGCGCAAGATTGAGGAGCTGAAACGTGGTTCGGGGCCCGGCAGCCCGGAAGATAAAATTGAAGAACTGAAAGAGAA
AAACTCTCAACTGAAGGAGAAGAACGAGGAGTTGAAACAAAAAATCTATGAGTTGAAGGAACTCGAGTAATGACCTAGG

Name: SBP1$_{9.a\text{-}L}$

DNA sequence:

TCTAGAAATAATTTTGTTTAACTTTAAGAAGGAGATATACACATGCATCACCATCACCACCATCACCATGAAAACCTCTATTT
TCAGGGTCATATGTCGCCTGAGGATGAAATACAGCAGTTGGAAGAGGAGATCTCCCAGCTTGAGCAAAAGAATAGCGAAC
TGAAAGAGAAGAACCAGGAACTGAAATATGGCAGTGGTCCAGGTTCCCCCGAGGACGAGAATGAGAAACTCGAAGAGAA
AATTTGGGAACTTAAACGTAAGAATGAGGAGCTAAAACGTGAAATAAAGGAGTTGGAAGAGGGAAGTGGACCAGGCGAC
ATCGAACAGGAGCTGGAAAGAGCTAAGCAGTCTATTGAGGAACTGGAGCGAGAAGTTAATCAGGAACGAAGCAGAATGC
AATACTTACAGACTAGGCTTTCCGGTTCAGGTCCCGGCTCACCTGAGGATAAGATCTCGCAGCTGAAAGAGAAAATCCAGC
AGCTGAAACAGGAAAACCAACAGCTTGAGGAAGAGAATAGTCAACTGGAATATGGATCAGGTCCCGGAAGTCCGGAAGA
CGAAAATGAGAAATTGGAACGTAAGAACGAAGAGCTTAAATGGGAAATCAAGAAGCTAGAGAGGGAGATAAAAGAGTTA
GAAAGAGGAAGCGGTCCTGGCTCCCCGGAAGACGAGATACGACAGTTGGAACAGGAGAATTCGCAGCTGGAGCGTGAAA
ACCAAAGACTAGAGCAAGAGATTTACCAATTGGAAAGAGGCTCGGGCCCCGGTGATATCGAACAGGAATTGGAACGTGCT
AAACAATCCATCGAGGAGTTGGAAAGGGAAGTCAATCAAGAAAGGTCTCGTATGCAATATTTACAAACTAGACTATCCGGA
TCGGGGCCTGGTAGCCCCGAGGATGAAATTAAGGAACTCGAATGGAAGAATGAGGAACTTAAAAGAGAAATCAAGGAACT
TGAGGAAAAGAACGAAGAGTTAAAACGAGGCTCAGGACCTGGCTCTCCTGAAGACAAAATTGAGGAACTGAAGGAGAA
GAACTCACAGTTGAAGGAAAAGAACGAAGAGTTGAAGCAGAAAATTTATGAGTTGAAGGAACTCGAGTAATGACCTAGG

Name: SBP2$_{9.a\text{-}L}$

DNA sequence:

TCTAGAAATAATTTTGTTTAACTTTAAGAAGGAGATATACACATGCATCACCATCACCACCATCACCATGAAAACTTGTATTT
TCAGGGTCATATGTCGCCTGAAGATGAGATCCAACAACTTGAGGAAGAGATTAGTCAGTTAGAGCAGAAGAATTCTGAGTT

AAAAGAGAAGAATCAGGAGCTTAAATATGGTTCAGGCCCCGGTTCTCCAGAAGATAAACTTGAGGAAATTAAGAGAGAAC
TTGAGGAGAATGAGTGGGAGCTGGAGAAAATTGAGAGAAAACTTGAGGAAAATAAACGTGGTTCTGGACCAGGTGACAT
TGAGCAGGAGCTGGAAAGGGCTAAGCAATCTATTGAGGAGCTGGAAAGGGAAGTGAATCAAGAACGATCCCGTATGCAAT
ACTTGCAGACGCGTTTGAGCGGGTCTGGACCTGGTAGTCCGGAAGACAAAATTTCTCAGCTTAAAGAGAAGATTCAACAA
CTGAAACAGGAGAATCAACAGCTGGAAGAGGAGAACTCTCAATTGGAGTACGGAAGTGGTCCGGGCTCTCCAGAGGACA
AAATAGAAGAACTCAAACGTGAGAACGAAGAGCTGGAATGGAAAATTGAAGAGCTTAAAAGAGAAAACGAGGAGCTGG
AAAAGGGTTCCGGGCCGGGATCTCCTGAGGATGAAATCAGGCAGTTGGAGCAGGAAAATTCACAATTGGAAAGAGAGAA
CCAAAGGCTTGAGCAGGAGATTTACCAGTTGGAACGTGGGTCTGGCCCAGGAGACATAGAGCAGGAACTAGAACGAGCA
AAGCAGAGTATAGAGGAATTAGAACGTGAAGTCAATCAAGAACGATCTCGTATGCAATACCTGCAAACACGACTGTCAGG
AAGTGGCCCAGGATCTCCCGAGGATAAGAACAAAGAACTCAAAGAGGAAAATAAAGAATTAGAATGGAAGATTGAGGAA
CTGAAGGAGAAAATTAAAGAACTGAAAGAGGGTTCCGGTCCCGGTTCTCCCGAGGATAAGATTGAGGAACTTAAAGAGAA
GAACTCACAGCTGAAGGAGAAGAACGAGGAGTTAAAGCAGAAGATCTATGAACTGAAAGAGCTCGAGTAATGACCTAGG

Name: SBP1$_{9.b}$

DNA sequence:

TCTAGATAATTTTGTTTAACTTTAAGAAGGAGATATACACCATGGGGCATCACCACCACCACCATCACCATATGGAAAACTTA
TATTTCCAAAGTGGTTCCGGGTCTCCTGAGGATGAGATCCGCCAGCTGGAGCAGGAAAACAGTCAGCTTGAACGCGAAAA
TCAGCGCCTTGAACAAGAGATCTACCAATTAGAGCGCGGTTCCGGGCCCGGGAGCCCGGAAGATGAAATTCAACAGCTTG
AGGAGGAAATCTCTCAGCTGGAACAAAAAAATTCCGAACTGAAAGAAAAAAATCAGGAGCTTAAATACGGTTCCGGCCCG
GGATCCCCAGAGGACGAAAATGAGAAGTTAGAACGCAAGAATGAGGAGTTGAAGTGGGAGATTAAGAAGCTTGAACGCG
AAATCAAAGAATTAGAACGCGGCAGCGGACCAGGGGACATTGAGCAAGAGCTTGAGCGCGCAAAGCAGTCCATTGAAGA
GTTGGAGCGCGAGGTAAACCAAGAACGTTCTCGCATGCAATATCTTCAAACCCGTTTGTCTGGGAGTGGACCGGGAAGCC
CTGAAGATAAGATTTCGCAATTGAAGGAAAAGATCCAACAACTGAAACAAGAGAATCAGCAACTTGAGGAGGAAAACTC
ACAACTTGAGTATGGTAGCGGGCCCGGCTCTCCTGAGGACGAGAACGAGAAATTAGAAGAGAAAATCTGGGAGCTTAAGC
GTAAAAATGAGGAGTTAAAGCGTGAAATCAAGGAATTAGAGGAAGGGTCCGGTCCAGGCAGTCCGGAGGATAAAATTGAA
GAATTAAAGGAGAAAAACAGTCAGTTGAAAGAAAAAAACGAGGAATTGAAACAAAAAATCTATGAGTTAAAGGAGGGCT
CTGGCCCAGGTGATATTGAACAGGAGTTGGAACGTGCCAAACAATCAATTGAAGAGCTGGAACGTGAGGTCAACCAGGAA
CGCAGCCGCATGCAGTATTTACAGACTCGTTTAAGCGGCTCCGGCCCCGGATCGCCTGAGGACGAAATCAAAGAGCTGGA
ATGGAAAAACGAGGAGCTGAAACGTGAAATTAAAGAATTGGAGGAGAAAAATGAAGAATTAAAACGCCTTGAACTCGAG
TAATGACCTAGG

Name: SBP2$_{9.b}$

DNA sequence:

TCTAGAATAATTTTGTTTAACTTTAAGAAGGAGATATACACCATGGGGCATCACCACCACCACCATCACCATATGGAAAACTT
ATATTTCCAAAGTGGTTCCGGGTCTCCTGAGGACAAGAACAAGGAACTGAAGGAAGAAAACAAAGAGCTTGAATGGAAA
ATCGAGGAATTGAAAGAGAAGATTAAGGAACTGAAAGAAGGGTCTGGGCCAGGGGATATTGAGCAAGAGCTTGAGCGTG
CCAAGCAGTCAATCGAAGAGCTTGAACGTGAAGTGAACCAGGAGCGCTCCCGTATGCAATATTTGCAGACGCGTCTGTCTG
GCAGTGGGCCCGGTTCTCCAGAGGACAAAATTTCGCAATTGAAAGAAAAAATCCAGCAGCTGAAACAAGAGAACCAACA
GTTAGAGGAGGAGAATTCCCAATTGGAATACGGGTCTGGTCCCGGTTCGCCCGAGGACAAAAACGAAGAATTAAAGCGCG
AGATCAAGGAGTTAGAGTGGGAGAACGAGGAACTGGAGCGCAAAATTGAAGAGCTGAAGCGTGGATCTGGGCCAGGGAG
TCCTGAAGATAAGATTGAAGAATTAAAGGAGAAAAACTCGCAGCTTAAGGAGAAAAATGAAGAACTGAAGCAGAAAATTT
ACGAACTGAAGGAGGGCTCGGGCCCGGGTGATATTGAGCAAGAACTGGAACGCGCTAAGCAATCTATTGAGGAACTTGAG
CGTGAGGTTAACCAGGAACGCTCTCGTATGCAGTACCTTCAAACCCGCTTGTCAGGTTCGGGCCCAGGCTCTCCTGAAGAC
AAAATCGAAGAACTTAAGCGCGAAAACGAGGAACTGGAGTGGAAGATCGAGGAATTGAAACGTGAGAATGAAGAATTGG
AGAAGGGCTCAGGGCCCGGATCCCCTGAAGATGAGATCCGCCAGTTAGAGCAGGAAAATAGCCAACTGGAGCGTGAGAAT
CAGCGCTTGGAGCAGGAGATTTACCAGTTGGAGCGCGGAAGCGGCCCAGGATCTCCAGAAGATGAAATTCAGCAACTTGA
GGAAGAGATCTCACAGTTAGAACAAAAGAATAGTGAGCTGAAGGAAAAAAATCAGGAGCTTAAGTATCTCGAGTAATGAC
CTAGG

Name: SBP1$_{11}$

DNA sequence:

TCTAGAAATAATTTTGTTTAACTTTAAGAAGGAGATATACACCATGGGGCATCACCACCACCACCATCACCATATGTCCCCAG
AAGACAAAAATAAAGAGCTGAAGGAGGAAAACAAGGAGTTAGAGTGGAAAATCGAGGAATTAAAGGAAAAAATTAAGG
AACTGAAGGAGGGTAGTGGACCGGGAAGTCCAGAAGATAAGATCGAGGAACTTAAACGCGAGAATGAAGAATTGGAGTG
GAAGATTGAGGAATTAAAGCGCGAAAATGAAGAGCTTGAGAAAGGTTCCGGCGAAAACTTATATTTCCAAAGTGGTTCCG

GGTCTCCTGAGGATGAGATCCGCCAGCTGGAGCAGGAAAACAGTCAGCTTGAACGCGAAAATCAGCGCCTTGAACAAGA
GATCTACCAATTAGAGCGCGGTTCCGGGCCCGGGAGCCCGGAAGATGAAATTCAACAGCTTGAGGAGGAAATCTCTCAGC
TGGAACAAAAAAATTCCGAACTGAAAGAAAAAAATCAGGAGCTTAAATACGGTTCCGGCCCGGGATCCCCAGAGGACGA
AAATGAGAAGTTAGAACGCAAGAATGAGGAGTTGAAGTGGGAGATTAAGAAGCTTGAACGCGAAATCAAAGAATTAGAA
CGCGGCTGCGGACCAGGGGACATTGAGCAAGAGCTTGAGCGCGCAAAGCAGTCCATTGAAGAGTTGGAGCGCGAGGTAA
ACCAAGAACGTTCTCGCATGCAATATCTTCAAACCCGTTTGTCTGGGAGTGGACCGGGAAGCCCTGAAGATAAGATTTCGC
AATTGAAGGAAAAGATCCAACAACTGAAACAAGAGAATCAGCAACTTGAGGAGGAAAACTCACAACTTGAGTATGGTAG
CGGGCCCGGCTCTCCTGAGGACGAGAACGAGAAATTAGAAGAGAAAATCTGGGAGCTTAAGCGTAAAAATGAGGAGTTA
AAGCGTGAAATCAAGGAATTAGAGGAAGGGTCCGGTCCAGGCAGTCCGGAGGATAAAATTGAAGAATTAAAGGAGAAAA
ACAGTCAGTTGAAAGAAAAAAACGAGGAATTGAAACAAAAAATCTATGAGTTAAAGGAGGGCTCTGGCCCAGGTGATATT
GAACAGGAGTTGGAACGTGCCAAACAATCAATTGAAGAGCTGGAACGTGAGGTCAACCAGGAACGCAGCCGCATGCAGT
ATTTACAGACTCGTTTAAGCGGCTCCGGCCCCGGATCGCCTGAGGACGAAATCAAAGAGCTGGAATGGAAAAACGAGGAG
CTGAAACGTGAAATTAAAGAATTGGAGGAGAAAAATGAAGAATTAAAACGCCTTGAACTCGAGTAATGACCTAGG

Name: SBP2₁₁

DNA sequence:

TCTAGAAATAATTTTGTTTAACTTTAAGAAGGAGATATACACCATGGGTTGGTCTCACCCACAGTTCGAGAAATCTGCTTCA
CCCGAGGACAAGAACAAGGAACTGAAGGAAGAAAACAAAGAGCTTGAATGGAAAATCGAGGAATTGAAAGAGAAGATT
AAGGAACTGAAAGAAGGGTCTGGGCCAGGGGATATTGAGCAAGAGCTTGAGCGTGCCAAGCAGTCAATCGAAGAGCTTG
AACGTGAAGTGAACCAGGAGCGCTCCCGTATGCAATATTTGCAGACGCGTCTGTCTGGCAGTGGGCCCGGTTCTCCAGAG
GACAAAATTTCGCAATTGAAAGAAAAAATCCAGCAGCTGAAACAAGAGAACCAACAGTTAGAGGAGGAGAATTCCCAATT
GGAATACGGGTCTGGTCCCGGTTCGCCCGAGGACAAAAACGAAGAATTAAAGCGCGAGATCAAGGAGTTAGAGTGGGAG
AACGAGGAACTGGAGCGCAAAATTGAAGAGCTGAAGCGTGGATCTGGGCCAGGGAGTCCTGAAGATAAGATTGAAGAAT
TAAAGGAGAAAAACTCGCAGCTTAAGGAGAAAAATGAAGAACTGAAGCAGAAAATTTACGAACTGAAGGAGGGCTCGGG
CCCGGGTGATATTGAGCAAGAACTGGAACGCGCTAAGCAATCTATTGAGGAACTTGAGCGTGAGGTTAACCAGGAACGCT
CTCGTATGCAGTACCTTCAAACCCGCTTGTCAGGTTGTGGCCCAGGCTCTCCTGAAGACAAAATCGAAGAACTTAAGCGCG
AAAACGAGGAACTGGAGTGGAAGATCGAGGAATTGAAACGTGAGAATGAAGAATTGGAGAAGGGCTCAGGGCCCGGATC
CCCTGAAGATGAGATCCGCCAGTTAGAGCAGGAAAATAGCCAACTGGAGCGTGAGAATCAGCGCTTGGAGCAGGAGATTT
ACCAGTTGGAGCGCGGAAGCGGCCCAGGATCTCCAGAAGATGAAATTCAGCAACTTGAGGAAGAGATCTCACAGTTAGAA
CAAAAGAATAGTGAGCTGAAGGAAAAAAATCAGGAGCTTAAGTATGGCTCAGGCGAGAATTTGTATTTTCAGAGCGGTAGT
GGGTCACCTGAAGATGAGAACGAAAAACTTGAACGTAAGAATGAGGAATTAAAATGGGAAATTAAAAAGTTAGAACGCGA
GATTAAAGAGTTGGAACGTGGTTCTGGCCCCGGTTCTCCGGAAGATGAGATTAAAGAATTAGAATGGAAAAATGAGGAACT
GAAACGCGAGATCAAGGAATTAGAAGAAAAGAACGAGGAACTGAAGAGTCTCGAGCACCACCATCACCACCATCACCAC
TAATGACCTAGG

Name: TEV protease

DNA sequence:

TAACTTTAAGAAGGAGATATACATATGGGCCATCACCATCATCATCACCATCATGGAGAAAGCTTGTTTAAGGGACCACGTG
ATTACAACCCGATATCGAGCACCATTTGTCACTTGACGAATGAATCTGATGGGCACACAACATCGTTGTATGGTATTGGATTT
GGTCCCTTCATCATTACAAACAAGCACTTGTTTAGAAGAAATAATGGAACACTGTTGGTCCAATCACTACATGGTGTATTCA
AGGTCAAGAACACCACGACTTTGCAACAACACCTCATTGATGGGAGGGACATGATAATTATTCGCATGCCTAAGGATTTCCC
ACCATTTCCTCAAAAGCTGAAATTTAGAGAGCCACAAAGGGAAGAGCGCATATGTCTTGTGACAACCAACTTCCAAACTAA
GAGCATGTCTAGCATGGTGTCAGACACCAGTTGCACATTCCCTTCATCTGATGGCATATTCTGGAAGCATTGGATTCAAACC
AAGGATGGGCAGTGTGGCAGTCCATTAGTATCAACTAGAGATGGGTTCATTGTTGGTATACACTCAGCATCGAATTTCACCA
ACACAAACAATTATTTCACAAGCGTGCCGAAAAACTTCATGGAATTGTTGACAAATCAGGAGGCGCAGCAGTGGGTTAGT
GGTTGGCGATTAAATGCTGACTCAGTATTGTGGGGGGGCCATAAAGTTTTCATGAGCAAACCTGAAGAGCCTTTTCAGCCA
GTTAAGGAAGCGACTCAACTCATGAGTGAATTGGTGTACTCGCAATAATAGCTCGAGTACCTAGGCTGCTAAACAAAGCCC
GAA
