# Supplementary material for: Self-assembly and regulation of protein cages from pre-organised coiled-coil modules
Source: Nat Commun. 2021 Feb 11;12:939. doi: 10.1038/s41467-021-21184-6 (PMC7878516; doi:10.1038/s41467-021-21184-6)
Supplement: Supplementary file 2 — Description of Additional Supplementary Files [file 41467_2021_21184_MOESM2_ESM.pdf]

Description of Additional Supplementary Files

File Name: Supplementary Software 1

Description: The source code of the software used for evaluating CCPO cage's topology and for designing molecular models is provided here. The software is distributed under the MIT license.
