# Supplementary material for: Self-assembly and regulation of protein cages from pre-organised coiled-coil modules
Source: Nat Commun. 2021 Feb 11;12:939. doi: 10.1038/s41467-021-21184-6 (PMC7878516; doi:10.1038/s41467-021-21184-6)
Supplement: Supplementary file 4 — Reporting Summary [file 41467_2021_21184_MOESM4_ESM.pdf]

nature research

Corresponding author(s): Roman Jerala

Last updated by author(s): Dec 21, 2020

Reporting Summary

Nature Research wishes to improve the reproducibility of the work that we publish. This form provides structure for consistency and transparency in reporting. For further information on Nature Research policies, see our Editorial Policies and the Editorial Policy Checklist.

Statistics

For all statistical analyses, confirm that the following items are present in the figure legend, table legend, main text, or Methods section.

n/a	Confirmed	
☐	☒	The exact sample size (*n*) for each experimental group/condition, given as a discrete number and unit of measurement
☐	☒	A statement on whether measurements were taken from distinct samples or whether the same sample was measured repeatedly
☒	☐	The statistical test(s) used AND whether they are one- or two-sided<br>*Only common tests should be described solely by name; describe more complex techniques in the Methods section.*
☒	☐	A description of all covariates tested
☒	☐	A description of any assumptions or corrections, such as tests of normality and adjustment for multiple comparisons
☐	☒	A full description of the statistical parameters including central tendency (e.g. means) or other basic estimates (e.g. regression coefficient) AND variation (e.g. standard deviation) or associated estimates of uncertainty (e.g. confidence intervals)
☒	☐	For null hypothesis testing, the test statistic (e.g. *F*, *t*, *r*) with confidence intervals, effect sizes, degrees of freedom and *P* value noted<br>*Give P values as exact values whenever suitable.*
☒	☐	For Bayesian analysis, information on the choice of priors and Markov chain Monte Carlo settings
☒	☐	For hierarchical and complex designs, identification of the appropriate level for tests and full reporting of outcomes
☒	☐	Estimates of effect sizes (e.g. Cohen's *d*, Pearson's *r*), indicating how they were calculated

Our web collection on statistics for biologists contains articles on many of the points above.

Software and code

Policy information about availability of computer code

Data collection	CD data were collected with the software Chirascan 4.5 (Applied Photophysics, UK).<br>SEC-MALS measurements were collected with Astra 7.0 software (Wyatt, CA USA).<br>SAXS data were integrated in the SASFLOW pipeline v 3.0 (PETRA III, DESY) (ref. 74).<br>ITC data were collected with the software VPViewer 1.4.12 (Malvern Panalytical, UK).<br>Negative stain EM image processing was performed using the Scipion platform v 1.0 (http://scipion.cnb.csic.es/).<br>Fuorescence spectra were recorded with the software Gen5 1.1. (BioTeK)
Data analysis	The CoCoPOD software v 0.0.1 (ref. 46) was expanded to allow for modelling of multi-chain CCPO cages and was used to design and model CCPO proteins. The updated source code is available with the current submission.<br>CD and ITC measurements were analysed with the model-fitting software glox v 0.2 described by Drobnak et al. (ref. 69).<br>Analysis of SAXS curves and ab initio modelling were performed using the ATSAS suite v. 2.8.0 (ref. 75).<br>SAXS profiles were calculated from molecular models and compared to experimental data using Pepsi-SAXS v 3.0 (ref. 76).<br>Internal cavities of ab initio models were evaluated with PyMOL Molecular Graphics System v 2.3 (plugin CAVER v 3.0).<br>Negative stain EM image processing was performed using the Scipion platform v 1.0 (http://scipion.cnb.csic.es/) (ref. 77) and Xmipp v 3.0 (https://github.com/I2PC/scipion-em-xmipp).<br>The amino acid contact map was generated using CMView v 1.1.1 (ref. 80).

For manuscripts utilizing custom algorithms or software that are central to the research but not yet described in published literature, software must be made available to editors and reviewers. We strongly encourage code deposition in a community repository (e.g. GitHub). See the Nature Research guidelines for submitting code & software for further information.

Data

Policy information about availability of data

All manuscripts must include a data availability statement. This statement should provide the following information, where applicable:

- Accession codes, unique identifiers, or web links for publicly available datasets
- A list of figures that have associated raw data
- A description of any restrictions on data availability

Source data are provided with this paper. EM data for BIP18SN have been deposited into the EM database EMDB (www.ebi.ac.uk/pdbe/emdb) with accession code EMD-11831 (https://www.ebi.ac.uk/pdbe/entry/emdb/EMD-11831). SAXS scattering data has been deposited into SASBDB (www.sasbdb.org) with accession codes: SASDJU5 for BIP18SN (https://www.sasbdb.org/data/SASDJU5/) and SASDJV5 for the complex SBP129.b (https://www.sasbdb.org/data/SASDJV5/).

Field-specific reporting

Please select the one below that is the best fit for your research. If you are not sure, read the appropriate sections before making your selection.

- [x] Life sciences
- [ ] Behavioural & social sciences
- [ ] Ecological, evolutionary & environmental sciences

For a reference copy of the document with all sections, see nature.com/documents/nr-reporting-summary-flat.pdf

Life sciences study design

All studies must disclose on these points even when the disclosure is negative.

Sample size	The sample size was chosen in accordance with previous experimental experience with other reported CCPO cages. Low-deviation between individual measurements suggested sample sizes were sufficient. No population-based studies were performed in this research. Statistical analyses performed are limited to averaging, standard deviation calculation, standard curve-fitting and data validation procedures.
Data exclusions	X-ray scattering frames that exhibited radiation damage were excluded in the data processing step as standard in SAXS analysis. Heat effect from the first ITC injection (2 µl) was not included in the fitting, the exclusion was pre-determined in base of previous experience with technical limitation of the instrument.
Replication	Reproducibility of SAXS data was confirmed by redundancy in the data collection (i.e. multiple frames per sample).<br>Biophysical analysis (CD, SEC-MALS, FRET, PAGE) was repeated at least two times on independent samples to ensure reproducibility. All the results were comparably successful and the data shown in the article is representative for all the attempts.
Randomization	No experiment required randomization since in all the experiments the samples were prepared, treated and analysed in the same manner.
Blinding	Blinding was not required for the experiments performed in this article since data collection and method of analysis were not affected by the knowledge of sample identity.

Reporting for specific materials, systems and methods

We require information from authors about some types of materials, experimental systems and methods used in many studies. Here, indicate whether each material, system or method listed is relevant to your study. If you are not sure if a list item applies to your research, read the appropriate section before selecting a response.

Materials & experimental systems

n/a	Involved in the study	
[x]	[ ]	Antibodies
[x]	[ ]	Eukaryotic cell lines
[x]	[ ]	Palaeontology and archaeology
[x]	[ ]	Animals and other organisms
[x]	[ ]	Human research participants
[x]	[ ]	Clinical data
[x]	[ ]	Dual use research of concern

Methods

n/a	Involved in the study	
[x]	[ ]	ChIP-seq
[x]	[ ]	Flow cytometry
[x]	[ ]	MRI-based neuroimaging
